# A baseline study of interpretable machine learning using GC-MS breath VOCs for classifying asthma, bronchiectasis, and COPD

**DOI:** 10.1038/s41598-025-28143-x

**Published:** 2025-12-23

**Authors:** Eun-Ji Ko, Si-On Bae, Daesung Kang

**Affiliations:** School of Bio-Health Convergence, College of Natural Sciences, Sungshin Women’s University, Seoul, Republic of Korea

**Keywords:** Breathomics,, Machine learning, Respiratory diseases classification, Shapley additive explanations (SHAP), Volatile organic compounds (VOCs), Biomarkers, Computational biology and bioinformatics, Diseases, Medical research

## Abstract

Accurate differentiation among asthma, bronchiectasis, and chronic obstructive pulmonary disease (COPD) remains a critical challenge due to overlapping clinical symptoms and limitations of conventional diagnostic tools. This study establishes a transparent, reproducible baseline using gas chromatography-mass spectrometry (GC-MS) data derived from exhaled breath to classify asthma, bronchiectasis, and COPD. Using a publicly available clinical dataset comprising 121 breath samples and 76 shared volatile organic compounds (VOCs), we evaluated seven supervised classifiers under nested cross-validation. Among the classifiers, XGBoost achieved the highest performance, with a mean accuracy of 95.83% and macro-averaged AUC of 0.998. To enhance clinical interpretability, we applied Shapley Additive exPlanations (SHAP) to identify the most influential VOCs for each disease class. This analysis revealed several candidate biomarkers with disease-specific or cross-disease relevance, such as 2-pentylfuran and hexadecane. This integrative approach demonstrates the potential of breathomics combined with explainable AI as a scalable and non-invasive tool for respiratory disease classification and biomarker discovery. By providing this reproducible baseline, our work offers a reference point for future methodological advances and clinical validation using breathomics data.

**Supplementary Information:**

The online version contains supplementary material available at 10.1038/s41598-025-28143-x.

## Introduction

Respiratory diseases such as asthma, bronchiectasis, and chronic obstructive pulmonary disease (COPD) are prevalent and heterogeneous conditions that often present with overlapping clinical symptoms. This diagnostic ambiguity poses a persistent challenge in pulmonary medicine, as timely and precise differentiation among these diseases is essential for effective treatment and management^1^. However, current diagnostic modalities including spirometry, radiological imaging, and symptom-based clinical assessments have significant limitations. Spirometry, while routinely used to assess lung function, often lacks the specificity required to distinguish among different respiratory conditions^2,3^. Radiological methods such as chest X-rays and CT scans can detect structural abnormalities but are limited in identifying early-stage disease or underlying biochemical changes^4^. Clinical assessments, though indispensable, are inherently subjective and prone to inter-practitioner variability^5^. Moreover, these conventional approaches are often time-consuming and insufficiently sensitive to the subtle metabolic changes that precede overt clinical manifestations, leading to misdiagnosis or delayed intervention^5,6^. Such diagnostic errors are well documented: Barthwal et al. reported that 11.1% of patients initially diagnosed with asthma and 72.5% of those labeled as COPD were later reclassified upon re-evaluation^7^. Similarly, up to 19.5% of new asthma cases have been retrospectively misclassified as COPD or emphysema, while COPD underdiagnosis and false diagnosis remain pervasive, with estimates ranging from 65 to 80% in population-based studies^8,9^.

In this context, breathomics has emerged as a promising non-invasive diagnostic tool that analyzes the molecular composition of exhaled breath. Specifically, it focuses on volatile organic compounds (VOCs), which are gaseous metabolites produced through endogenous metabolic activity, inflammatory responses, oxidative stress, and interactions with the microbiome. These VOCs reflect both local airway and systemic physiological states^10,11^. The ability to capture these molecules in real time without the need for invasive sampling makes breathomics especially attractive for disease diagnosis and monitoring. Compared to other biological samples such as blood, urine, or saliva, exhaled breath offers greater accessibility and patient comfort, making it particularly suitable for frequent sampling in clinical settings^12,13^.

Among the analytical platforms employed in breathomics, gas chromatography-mass spectrometry (GC-MS) remains the gold standard in breathomics due to its high sensitivity and resolution in profiling VOCs, as well as its relative affordability, accessibility, and widespread availability in clinical and research laboratories^14,15^. Despite its analytical strengths, GC-MS generates complex and high-dimensional data that necessitate advanced computational methods to derive meaningful clinical insights^6,16^. Furthermore, breathomics studies often involve relatively small cohorts, which amplifies the analytical challenges and increases the risk of overfitting in statistical modeling^17^.

To address these issues, computational methods based on machine learning have proven effective for extracting patterns, performing classification, and identifying candidate biomarkers in omics datasets. Machine learning algorithms are well-suited for analyzing breathomics data, as they can handle multivariate inputs, model nonlinear interactions, and yield robust performance even in datasets of limited size^15,18^. Additionally, recent advances in explainable artificial intelligence (XAI), such as SHapley Additive exPlanations (SHAP), provide interpretable insights into model behavior and enable the identification of features most critical for disease discrimination^4,19^. XAI is broadly defined as a set of methodologies designed to render the output and internal decision-making process of complex machine learning models transparent, interpretable and understandable to human users. These methods can bridge the gap between predictive accuracy and clinical interpretability, which is essential for translational applications in healthcare.

In this study, we utilize a recently published and openly available clinical breathomics dataset, which includes GC-MS-based VOC profiles collected from patients diagnosed with asthma, bronchiectasis, and COPD^20^. We focused on VOC features that were consistently detected across all three disease groups and applied a supervised machine learning framework for disease classification. Furthermore, we employed SHAP analysis to interpret model predictions and identify VOCs most relevant to each disease. It is worth noting that this dataset was originally released by Kuo et al. as a data descriptor^20^. Their work emphasized standardized data collection and technical validation, but did not perform predictive modeling or biomarker interpretation. Our study builds upon this foundation by establishing baseline machine learning performance and interpretable analyses. Specifically, we employed multiple supervised machine learning classifiers with rigorous nested cross-validation and SHAP-based interpretability to ensure robust and transparent evaluation, with full methodological details provided in Methods Sect. Classification models and Model interpretation.

This study establishes a transparent, reproducible baseline on this dataset and, for the first time to our knowledge, apply SHAP to provide clinically interpretable insights into disease-specific VOC patterns.

This work demonstrates the feasibility and effectiveness of combining breathomics with interpretable machine learning models to improve the classification of clinically overlapping respiratory diseases. Moreover, it offers insights into the most informative VOC biomarkers, thereby contributing to the broader goal of personalized and early diagnosis in pulmonary medicine. Our contributions are as follows:

- We provide a transparent and reproducible baseline for classifying respiratory diseases based on GC-MS-derived breath VOCs.
- We apply SHAP-based explainability to derive clinically interpretable VOC attributions that distinguish asthma, bronchiectasis, and COPD.
- We support the clinical potential of breathomics as a scalable and non-invasive diagnostic tool for respiratory disease stratification.

## Related works

The analysis of exhaled breath as a non-invasive diagnostic approach has gained increasing attention over the past decade, particularly for respiratory diseases. The field of breathomics, which focuses on the analysis of VOCs in exhaled breath, has been explored in various clinical applications including asthma, bronchiectasis, COPD, and lung cancer. VOCs are chemically diverse metabolites that reflect underlying inflammatory, metabolic, or microbial processes, and they offer promising potential as disease-specific biomarkers^6,21,22^.

### Breathomics-based VOC profiling for asthma diagnosis and phenotyping

Asthma is a prevalent chronic inflammatory airway disease that remains difficult to diagnose and monitor accurately, especially in pediatric populations. Traditional diagnostic tools such as spirometry, FENO, and sputum cytology are either invasive or require significant patient cooperation, making them less suitable for children or for longitudinal monitoring^17^. In this context, exhaled VOCs have emerged as promising non-invasive biomarkers that reflect airway inflammation, oxidative stress, and lipid peroxidation.

A systematic review and meta-analysis by Cavaleiro et al. reported a pooled AUC of 0.94 for asthma detection using exhaled VOC profiles, underscoring their strong diagnostic potential^23^. However, only a small fraction of the included studies performed external validation, emphasizing the need for robust and reproducible study design before clinical translation.

In pediatric asthma, breathomics shows considerable promise. Neerincx et al. reviewed the current status of VOC-based diagnostics for childhood asthma and concluded that most existing studies reported moderate to excellent prediction accuracy (80–100%), typically using 6–28 VOCs^17^. However, they emphasized the need for standardized sampling protocols, external validation, and longitudinal studies to establish clinical utility. Smolinska et al. conducted a prospective study on 252 preschool-aged children, demonstrating that a panel of 17 VOCs could predict future asthma development with 80% accuracy in an independent test set^22^. These VOCs were associated with oxidative stress and inflammatory processes, suggesting a biochemical basis for early detection.

From a phenotyping perspective, Suzukawa et al. analyzed exhaled VOCs from 245 patients with severe asthma and identified distinct chemical profiles across five phenotypic clusters^24^. Although statistical significance was limited after FDR correction, their findings suggest that VOC signatures may help differentiate between early-onset and late-onset asthma subtypes.

Alternative analytical platforms such as electronic noses (eNoses) have also been applied to asthma subtyping. Abdel-Aziz et al. evaluated eNose breath profiles from over 650 participants across four independent cohorts and used machine learning models to distinguish atopic from non-atopic asthma^25^. Their classifiers achieved AUCs ≥ 0.84 in training and ≥ 0.72 in external validation, demonstrating robust generalization. Notably, unsupervised Bayesian network analysis confirmed that the eNose signatures for atopy were not confounded by other clinical variables.

Collectively, these studies represent the potential of breathomics to transform asthma diagnostics and phenotyping through non-invasive, rapid, and biologically informative measurements.

### Diagnostic and prognostic utility of VOCs in COPD

Multiple studies have investigated the utility of exhaled VOCs as non-invasive biomarkers for diagnosing COPD, detecting exacerbations, and stratifying patient phenotypes. A prospective follow-up study by van Velzen et al. demonstrated that breath profiles acquired via GC-MS and eNose could distinguish between stable COPD, exacerbation, and recovery phases, with classification accuracies of 71% and 78%, respectively. This provides proof of principle for using breath VOCs as dynamic markers of disease activity^26^.

Building on this, van Poelgeest et al. conducted a systematic review and validation study that identified and confirmed six VOCs significantly associated with COPD exacerbations. Using sparse partial least squares-discriminant analysis on longitudinal data from the TEXACOLD cohort, the composite model achieved an AUC of 0.98, diagnostic accuracy of 94.3%, sensitivity of 97%, and specificity of 93%, indicating strong potential for breath-based monitoring devices^27^.

VOC-based breathomics also shows promise in differentiating COPD subgroups. Basanta et al. applied gas chromatography time-of-flight mass spectrometry (GC-ToF-MS) and multivariate modeling to classify COPD patients with clinical features such as sputum eosinophilia and frequent exacerbations. Their models achieved AUCs up to 0.94–0.95 for subgroup identification and revealed VOC signatures correlated with inflammatory markers and exacerbation history^28^.

In a larger machine learning-based study by Phillips et al., VOC profiles from 119 COPD patients and 63 matched controls were analyzed. The resulting models achieved a classification accuracy of 79% and an AUC of 0.82. Importantly, smoking status was found to significantly influence performance, emphasizing the need to control for this confounder in future analyses^29^.

Lastly, Binson et al. introduced a portable, low-cost e-nose system integrated with ensemble learning algorithms, including extreme gradient boosting (XGBoost), to distinguish COPD and lung cancer from healthy controls. The model attained classification accuracies of 76.67% for COPD and 79.31% for lung cancer, underscoring the clinical viability of mobile diagnostic technologies^30^.

Together, these studies affirm the diagnostic value of exhaled VOCs in COPD, both for differentiating disease states and for identifying clinically relevant phenotypes.

### Breathomics for diagnosis and phenotyping of bronchiectasis

While breathomics research in bronchiectasis is less extensive compared to asthma and COPD, emerging studies demonstrate its growing potential in diagnosis, phenotyping, and disease monitoring. In a recent study by Gu et al., VOCs in exhaled breath condensate were profiled using solid-phase microextraction gas chromatography-mass spectrometry (SPME-GC-MS) to differentiate stable bronchiectasis patients based on hypoxia status and *Pseudomonas aeruginosa* infection. Specific compounds such as 10-heptadecenoic acid, heptadecanoic acid, longifolene, and decanol were significantly elevated in hypoxic patients, while other metabolites (e.g., 13-octadecenoic acid, phenol, pentadecanoic acid) were associated with *P. aeruginosa* positivity. Notably, 10-heptadecenoic acid was identified as an independent prognostic marker for hypoxia severity in multivariate analysis, suggesting a link between breath VOCs and bronchiectasis pathophysiology^31^.

Complementing this, Fan et al. conducted a large-scale cross-sectional study using high-pressure photon ionization time-of-flight mass spectrometry (HPPI-TOF-MS) on exhaled breath from 215 bronchiectasis patients and 295 controls. A machine learning-based diagnostic model trained on the top ten breath biomarkers achieved an AUC of 0.94, sensitivity of 90.7%, specificity of 85%, and overall accuracy of 87.4%. Furthermore, several breath biomarkers were associated with clinical parameters such as disease stage (acute vs. stable), hemoptysis, *P. aeruginosa* or *nontuberculous mycobacterium* infection, number of affected lobes, and lung function indices—supporting the role of breathomics in both diagnosis and individualized patient stratification^32^.

Broader insight into breath analysis for pulmonary exacerbations in mucociliary clearance disorders, including bronchiectasis, was provided by a systematic review by Nessen et al. The review encompassed 18 studies (primarily on cystic fibrosis and primary ciliary dyskinesia) and highlighted hydrocarbons, particularly alkenes and pentane, as potential biomarkers. However, heterogeneity in experimental design, exacerbation definitions, and analytical platforms limited replicability across studies, indicating a need for standardized protocols and longitudinal validation^33^.

### Machine learning applications for VOC-based respiratory disease classification

Recent advancements in machine learning have accelerated the application of breathomics for respiratory disease classification. These efforts leverage the high-dimensional and complex nature of exhaled VOCs and demonstrate the feasibility of non-invasive diagnostic modeling across a variety of pulmonary conditions.

In a large-scale study targeting pulmonary tuberculosis (PTB), Fu et al. collected breath samples from 518 PTB patients and 887 controls using HPPI-TOF-MS. Using ensemble models such as XGBoost and random forest, the researchers achieved high performance in both validation and blinded test sets, with an AUC of 0.975, accuracy of 92.6%, and specificity of 93.0%, representing the robustness and scalability of breath-based machine learning models in infectious respiratory diseases^34^.

For interstitial lung disease (ILD) classification, Massenet et al. analyzed breath VOC profiles from patients with systemic sclerosis (SSc) and systemic sclerosis-associated ILD (SSc-ILD) using thermal desorption comprehensive two-dimensional gas chromatography high-resolution mass spectrometry (TD-GC×GC-HRMS). From ~ 800 detected features, a partial least squares-discriminant analysis (PLS-DA) model identified nine discriminative VOCs and achieved an AUC of 0.82, sensitivity of 77%, and specificity of 100%. Importantly, the study demonstrated the feasibility of multicentric breathomics protocols and linked VOC profiles to pulmonary function metrics such as DLCO, reinforcing the physiological relevance of the predictive markers^35^.

Most directly relevant to our study, Tian et al. developed classification models for COPD, asthma, and preserved ratio impaired spirometry (PRISm) using VOCs captured via portable micro gas chromatography. Involving 367 patients across multiple disease groups, the study identified specific VOC markers for differentiating COPD vs. asthma, PRISm vs. healthy, and other pairwise comparisons. Machine learning algorithms including random forest, support vector machine (SVM), and XGBoost were trained on both VOC features and clinical metadata. The optimal models yielded high classification accuracies across all disease pairs, illustrating the potential of breathomics in resolving clinically overlapping respiratory conditions^36^.

Taken together, these studies exemplify how machine learning synergizes with breath-based metabolomics to offer accurate, interpretable, and scalable diagnostic frameworks for respiratory diseases.

In this study, we build upon these previous findings by applying multiple machine learning classifiers to the open-access clinical breathomics dataset by Kuo et al. and conducting SHAP-based interpretation to identify disease-specific VOCs^20^. Our approach contributes to the growing field of interpretable machine learning in clinical breathomics and supports the development of accurate, non-invasive tools for respiratory disease classification.

## Results

### Performance comparison of machine learning models on breathomics data

Before presenting the classification results, we briefly summarize the dataset used in this study. The cohort comprised 121 exhaled breath samples, including 53 from asthma, 35 from bronchiectasis, and 33 from COPD patients. To enable fair comparison across disease groups, only 76 VOC features commonly detected in all three groups were retained after removing duplicated entries and harmonizing metabolite identifiers. These curated VOC intensity profiles, rather than raw peak tables, served as the standardized input for all machine learning experiments.

To compare the classification performance across various models, we employed a 5-fold nested cross-validation framework, which provides an unbiased estimate of generalization by ensuring that hyperparameter tuning and model evaluation are conducted on strictly separated data partitions. Performance metrics including accuracy, AUC, precision, sensitivity, and F1-score were computed for each model and summarized in Table 1 as mean ± standard deviation across outer folds.

As shown in Table 1, XGBoost consistently outperformed all other models, achieving the highest classification performance across all evaluation metrics, including accuracy (95.83%), AUC (0.998), precision (0.957), sensitivity (0.951), and F1-score (0.952). Random forest also showed strong results (accuracy = 90.90%, AUC = 0.982, F1-score = 0.891), followed by decision tree, logistic regression, and SVM, which delivered competitive yet slightly lower performance. On the other hand, k-nearest neighbors (kNN) and naïve Bayes exhibited considerably lower performance across all metrics, representing the superiority of ensemble-based methods in handling breathomics data for respiratory disease classification.

**Table 1:** Performance comparison of machine learning models for respiratory disease classification. Results represent the mean ± standard deviation of macro-averaged metrics across outer folds of 5-fold nested cross-validation. (Abbreviations: kNN = k-nearest neighbors, LR = logistic regression, NB = naïve Bayes, DT = decision tree, SVM = support vector machine, RF = random forest, and XGBoost = extreme gradient boosting.).

Models	Accuracy (%)	AUC	Precision	Sensitivity	F1-score
KNN	62.73 (8.16)	0.757 (0.064)	0.595 (0.084)	0.586 (0.080)	0.585 (0.079)
LR	89.17 (8.58)	0.956 (0.054)	0.887 (0.094)	0.879 (0.092)	0.879 (0.097)
NB	79.30 (5.99)	0.909 (0.078)	0.792 (0.082)	0.762 (0.066)	0.751 (0.061)
DT	90.00 (6.77)	0.924 (0.017)	0.889 (0.086)	0.881 (0.083)	0.879 (0.084)
SVM	82.63 (3.16)	0.946 (0.038)	0.814 (0.051)	0.805 (0.043)	0.801 (0.043)
RF	90.90 (3.14)	0.982 (0.017)	0.905 (0.037)	0.892 (0.035)	0.891 (0.034)
XGBoost	95.83 (4.56)	0.998 (0.004)	0.957 (0.049)	0.951 (0.052)	0.952 (0.051)

To further visualize the models’ discriminative capabilities, macro-average ROC curves were plotted in Fig. 2 A using aggregated outer-fold predictions. Here, the macro-average ROC was obtained by averaging the class-specific ROC curves across the three diseases, ensuring equal contribution of each class regardless of sample size. Consistent with the results in Table 1, XGBoost (AUC = 0.998) produced the most favorable ROC curve, confirming its excellent discrimination power among the three disease classes. Random forest (AUC = 0.982) and logistic regression (AUC = 0.956) also showed strong predictive power. In contrast, kNN (AUC = 0.757) demonstrated relatively poor discriminative ability. While the AUC values of the top-performing models were all relatively high (> 0.90), XGBoost and RF achieved superior performance on macro-averaged F1-score, precision, and sensitivity, showing their robustness under class imbalance.

Additionally, class-wise ROC curves were generated using the one-vs-rest approach across all models. As illustrated in Figure S1, XGBoost achieved near-perfect classification performance for all three diseases, with AUCs of 1.000 (asthma), 0.994 (bronchiectasis), and 0.995 (COPD).

**Fig. 1:**
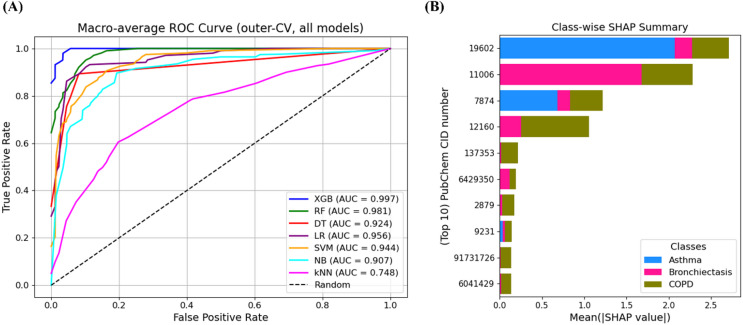
Performance and interpretability of machine learning models applied to breathomics data. (**A**) Macro-averaged ROC curves for seven classification models using outer cross-validation. (**B**) SHAP summary plot showing the top 10 VOCs contributing to classification across asthma, bronchiectasis, and COPD. Each horizontal bar represents a VOC, identified by its PubChem CID number on the y-axis, with bar length and color indicating the magnitude and class-specific contribution. (Abbreviations: kNN = k-nearest neighbors, LR = logistic regression, NB = naïve Bayes, DT = decision tree, SVM = support vector machine, RF = random forest, and XGBoost = extreme gradient boosting.)

For robustness, we additionally performed nonparametric bootstrapping (1,000 resamples) using the final refitted models to estimate 95% confidence intervals for all evaluation metrics. As shown in Table 1, the relatively small standard deviations across outer folds indicate stable performance across different train/test splits, while the bootstrap-based intervals in Table S1 provide an additional quantification of uncertainty. These bootstrap estimates differ slightly from those in Table 1 because Table 1 reflects variability across outer folds of nested cross-validation, whereas Table S1 captures resampling uncertainty around the fixed refitted model. Together, these complementary analyses support both the stability of the models and their generalizability within this dataset, while noting that external validation is required to establish true generalizability.

**Fig. 2:**
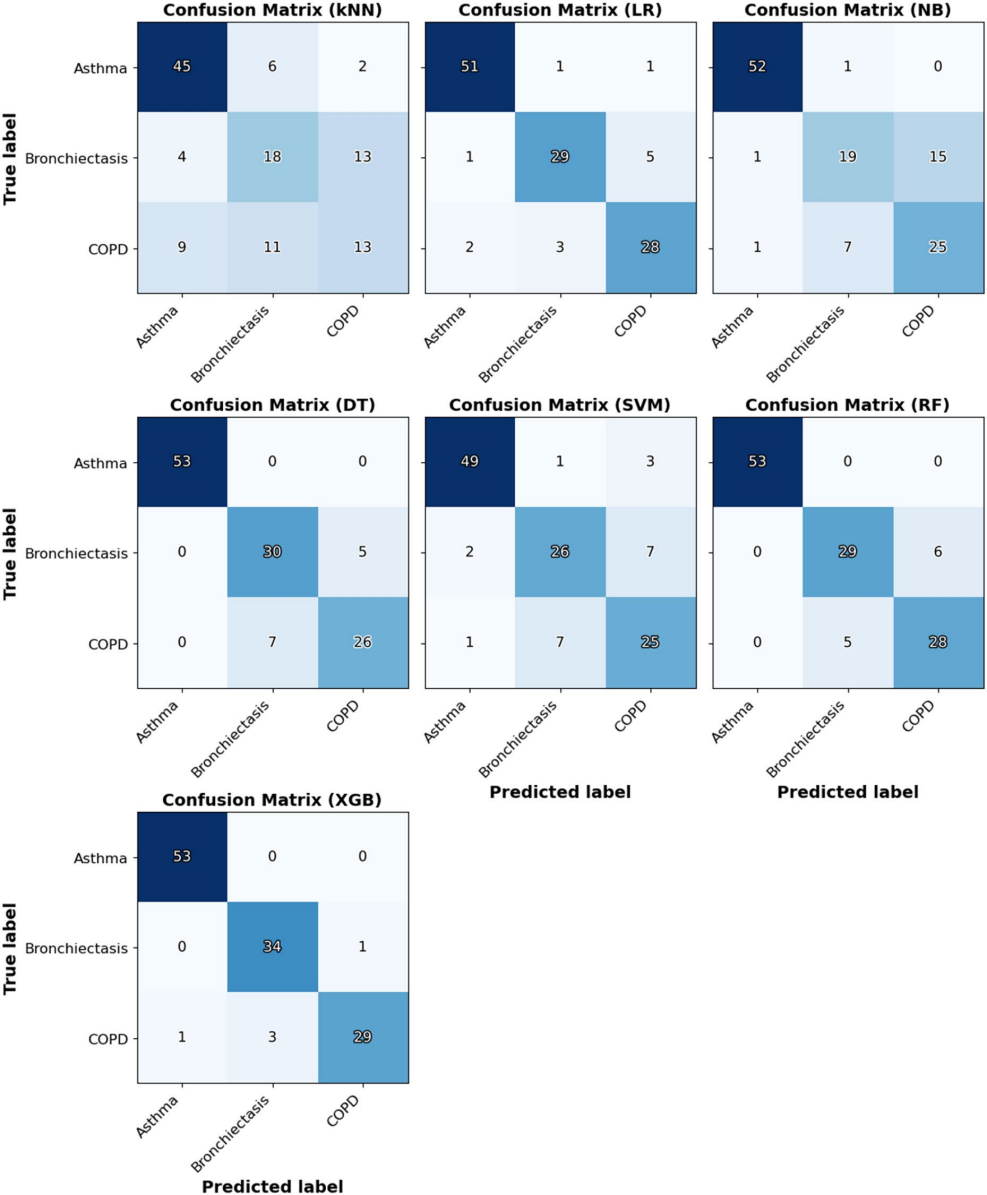
Confusion matrices for the seven classification models applied to breathomics data using outer cross-validation. (Abbreviations: kNN = k-nearest neighbors, LR = logistic regression, NB = naïve Bayes, DT = decision tree, SVM = support vector machine, RF = random forest, and XGBoost = extreme gradient boosting.).

To further illustrate the class-wise predictive performance of each model, confusion matrices from the outer cross-validation predictions are presented Fig. 2. Consistent with the quantitative metrics in Table 1, ensemble models such as random forest and XGBoost showed the most accurate classification across all three diseases, with XGBoost achieving nearly perfect separation (53/53 asthma, 34/35 bronchiectasis, and 29/33 COPD correctly classified). Decision tree and logistic regression also demonstrated strong performance, whereas kNN and naïve Bayes misclassified a larger number of bronchiectasis and COPD cases.

Table 2 summarizes the optimal hyperparameters selected via nested cross-validation for each classifier. For algorithms such as naïve Bayes and SVM, a clear consensus was observed across all outer folds, whereas kNN and decision tree exhibited co-modal outcomes with two competing parameter sets. Random forest and XGBoost showed no consensus, with heterogeneous parameter choices across folds, reflecting the limited sample size and high dimensionality of the dataset.

**Table 2:** Optimal hyperparameters selected via nested cross-validation across models. (Abbreviations: kNN = k-nearest neighbors, LR = logistic regression, NB = naïve Bayes, DT = decision tree, SVM = support vector machine, RF = random forest, and XGBoost = extreme gradient boosting. “Consensus” refers to whether a single hyperparameter setting dominated across all outer folds. “Co-modal” indicates two competing settings appeared with comparable frequency, while “No consensus” indicates heterogeneous selections without a clear dominant choice.).

Models	Most frequently selected hyperparameters	Consensus
kNN	n_neighbors = 3 (selected in 3 folds)	Co-modal
	n_neighbors = 15 (selected in 2 folds)	
LR	C = 1 (selected in 2 folds)	No consensus
	C = 10 (selected in 2 folds)	
	C = 100 (selected in 1 fold)	
NB	Default parameters (GaussianNB)	Consensus
DT	max_depth = 5; min_samples_split = 2 (selected in 4 folds)	Co-modal
	max_depth = 5; min_samples_split = 10 (selected in 1 folds)	
SVM	C = 1; Kernel = linear; Degree = 2 (selected in 5 folds)	Consensus
RF	max_depth = 10; min_samples_split = 5; n_estimators = 200	No consensus
	max_depth = 10; min_samples_split = 10; n_estimators = 200	
	max_depth = 10; min_samples_split = 10; n_estimators = 50	
	max_depth = 5; min_samples_split = 15; n_estimators = 200	
	max_depth = 5; min_samples_split = 2; n_estimators = 100	
XGBoost	n_estimators = 100; max_depth = 3; learning_rate = 0.01; subsample = 0.6; colsample_bytree = 0.8	No consensus (per-fold), Final model parameters shown
	n_estimators = 50; max_depth = 6; learning_rate = 0.1; subsample = 0.8; colsample_bytree = 0.8	
	n_estimators = 200; max_depth = 3; learning_rate = 0.2; subsample = 0.6; colsample_bytree = 1	
	n_estimators = 100; max_depth = 3; learning_rate = 0.1; subsample = 0.8; colsample_bytree = 0.8	
	n_estimators = 500; max_depth = 3; learning_rate = 0.01; subsample = 0.6; colsample_bytree = 0.6	

### Model interpretability via SHAP-based feature analysis

For SHAP-based interpretation, we refitted the XGBoost model on the entire dataset using the best parameters derived from full inner cross-validation (n_estimators = 200, max_depth = 6, learning_rate = 0.1, subsample = 0.6, colsample_bytree = 0.8). This ensured that SHAP explanations were derived from a stable refit model rather than one tied to a particular outer fold.

To elucidate the decision-making process of the best-performing classifier (XGBoost), we employed SHAP analysis that provides consistent and locally accurate feature attributions for complex machine learning models. SHAP values quantify the contribution of each VOC to the model’s predictions across all disease classes.

Figure 2B presents a SHAP summary plot showing the mean absolute SHAP values of the top 10 most influential VOCs. Each horizontal bar represents a VOC, identified by its PubChem CID number on the y-axis. The length and color of each segment indicate the magnitude and class-specific contribution of the VOC to the model’s output. Specifically, blue, pink, and olive-green colors show SHAP contributions to asthma, bronchiectasis, and COPD, respectively. For clarity, the same SHAP summary plot is reproduced in Supplementary Figure S3 with compound labels replaced by their IUPAC names instead of PubChem CIDs.

The SHAP summary plot depicts several key VOCs with strong influence on classification performance. Notably, CID 19,602 showed strongest contribution to asthma classification, with additional though smaller influence on COPD, and limited effect on bronchiectasis. This pattern suggests that while CID 19,602 is not a universal marker across all three diseases, it may serve as a shared but predominantly asthma-related feature. CID 11,006 had dominant influence on bronchiectasis predictions, while CIDs 7874 and 12,160 contributed meaningfully to COPD classification. Other features (CIDs 137353, 6429350, 2879 and 9231) exhibited class-specific contributions, emphasizing the ability of the model to capture biologically meaningful disease-specific chemical signatures in breathomics data. The PubChem CIDs and corresponding IUPAC names of the key compounds visualized in Fig. 2B are listed in Table 3.

**Table 3:** PubChem CID numbers and IUPAC names of the top 10 VOCs identified as important features for respiratory disease classification.

PubChem CID	IUPAC names
19,602	2-pentylfuran
11,006	hexadecane
7874	2,2,4,4,6,6,8,8,10,10,12,12,14,14-tetradecamethyl-1,3,5,7,9,11,13-heptaoxa-2,4,6,8,10,12,14-heptasilacyclotetradecane
12,160	1-ethyl-4-methylbenzene
137,353	3-ethyl-3-[4-(hydroxyamino)phenyl]piperidine-2,6-dione
6,429,350	1-[(1R,7R)−4-methylidene-7-propan-2-yl-1,2,3,3a,5,6,7,7a-octahydroinden-1-yl]ethanone
2879	4-methylphenol
9231	Azulene
91,731,726	(4-trimethylsilyloxybenzoyl) 4-trimethylsilyloxybenzoate
6,041,429	E)−3-(2,4-dimethoxyphenyl)−1-(2-hydroxyphenyl)prop-2-en-1-one

To further investigate class-specific feature contributions, Figure S2 provides a full view of the SHAP importance values of all 76 shared VOCs, separately for each disease class. These bar plots offer a more granular understanding of how each compound contributes to the model’s output in asthma, bronchiectasis, and COPD, extending the insights from the top-10-focused summary plot. The same plots with compound labels replaced by their IUPAC names are provided in Supplementary Figure S4.

**Fig. 3:**
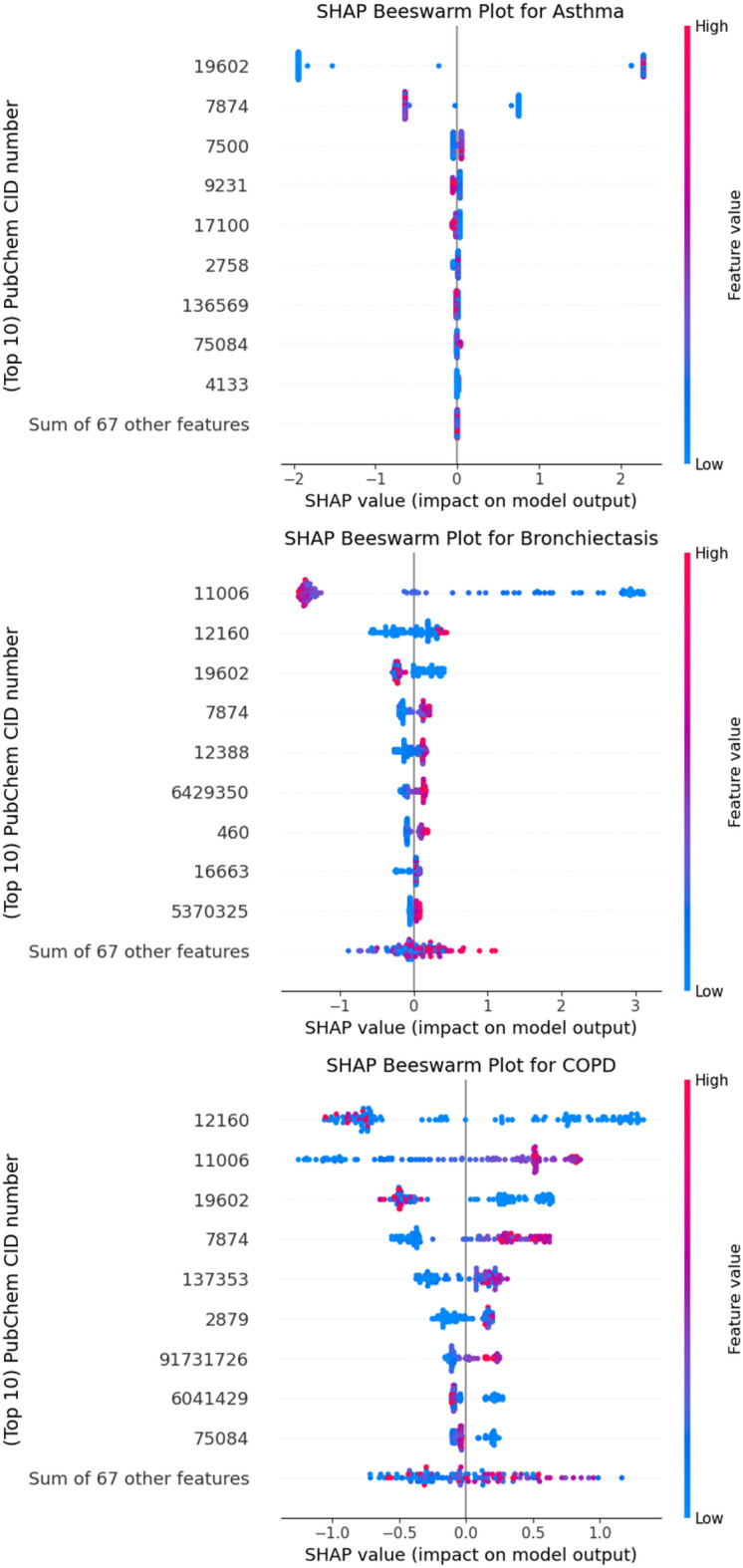
Class-wise SHAP beeswarm plots for asthma, bronchiectasis, and COPD. Each plot displays the 9 most influential VOCs for that disease, along with the summed contribution of all remaining 67 VOCs. Each point corresponds to one patient sample, with horizontal position indicating the SHAP value (impact on model output). Positive SHAP values indicate that a VOC increases the likelihood of the disease prediction, whereas negative values decrease it. Color represents the relative feature value for that sample (red = high, blue = low).

Further analysis of disease-specific predictions was conducted through SHAP beeswarm plots as shown in Fig. 3. These plots visualize the distribution of SHAP values for the top 9 VOCs per disease, capturing both the magnitude and direction of feature impact across individual samples. In asthma, CID 19,602 emerged as the most prominent contributor, consistently showing positive impact in samples with elevated concentrations. For bronchiectasis, CIDs 11,006 dominated feature impact, alongside contributions from CIDs 12,160, 19,602, and 7874. COPD predictions revealed a more distributed pattern, with CIDs 12,160, 11,006, 19,602, and 7874 all contributing substantially. The same beeswarm plots with compound labels replaced by their IUPAC names are provided in Supplementary Figure S5.

This SHAP-based interpretability analysis not only validates the learned patterns of the model but also allows for the identification of potential disease biomarkers. Several VOCs demonstrated clear discriminative power between asthma, bronchiectasis, and COPD, thereby enhancing the clinical relevance of breathomics-based disease classification.

## Discussion

This study extends prior evidence on the feasibility of supervised machine learning in breathomics by providing the first systematic multi-class baseline analysis of asthma, bronchiectasis, and COPD. Ensemble algorithms such as XGBoost and random forest achieved the strongest performance, reaffirming their robustness in handling high-dimensional VOC data even under limited sample conditions. These results highlight the potential of breathomics as a non-invasive diagnostic approach. Their superior performance can be attributed to the characteristics of the dataset: relatively small sample size (121 patients), high dimensionality (76 VOC features), and complex, potentially nonlinear metabolite—disease relationships. Ensemble methods such as random forest and XGBoost are particularly well-suited to these conditions because they reduce variance by aggregating multiple learners, capture nonlinear interactions through decision tree ensembles, and incorporate built-in mechanisms for regularization and feature subsampling^37,38^. Together, these properties mitigate overfitting and enhance predictive accuracy, explaining their robustness and superiority compared to simpler classifiers in this breathomics classification task.

Beyond predictive performance, the integration of SHAP-based interpretability enabled us to identify VOCs most influential to disease discrimination. Compounds such as CID 19,602 contributed most strongly to asthma, with additional but smaller influence on COPD and minimal effect on bronchiectasis, aligning with the class-wise SHAP patterns in Fig. 1(B). In contrast CID 11,006 was particularly influential in distinguishing bronchiectasis. These interpretable findings move beyond accuracy alone by offering biologically plausible insights that can guide further biochemical validation and biomarker discovery.

In addition to their statistical relevance, several of the SHAP-identified VOCs have literature support in disease-specific contexts. For instance, 2-pentylfuran (CID 19602) has been repeatedly detected in the breath of patients with chronic pulmonary disease colonized or infected by *Aspergillus fumigatus*, including asthma cohorts, which provides a biologically plausible link to eosinophilic airway inflammation and fungus-associated asthma endotypes^12^. In addition, although bronchiectasis-specific reports remain limited, hexadecane (CID 11006) has been observed among hydrocarbon signals associated with airway inflammation, suggesting a putative role that warrants bronchiectasis-specific validation^39^. For COPD, hydrocarbons such as hexadecane (CID 11006) and aromatic compounds like 1-ethyl-4-methylbenzene (CID 12160) have been described in breath or population studies, reinforcing their relevance^18,39^. Moreover, 2-pentylfuran (CID 19602) has also appeared in breath-based panels predicting COPD outcomes, underscoring its cross-disease importance^40^.

Beyond methodological considerations, the rationale for employing GC-MS breathomics lies in its unique diagnostic potential. GC-MS provides high sensitivity and specificity for volatile organic compounds, enabling reliable detection of trace metabolites associated with airway inflammation, oxidative stress, and microbial processes^41^. Compared to blood or urine assays, exhaled breath can be collected rapidly, repeatedly, and non-invasively, minimizing patient burden. These properties make GC-MS breathomics especially advantageous for scalable, real-time disease monitoring in respiratory medicine, and support its application in classification tasks targeting clinically overlapping phenotypes.

Compared to previous studies in breathomics, our work offers several novel contributions in terms of disease coverage, systematic and reproducible design, and interpretability. Kuo et al., who originally published the dataset used in this study, primarily presented exploratory visualizations using unsupervised clustering approaches, such as correlation-based heatmaps combined with single-linkage hierarchical clustering^20^. In contrast, our study is the first, to our knowledge, to perform a systematic supervised learning analysis using this dataset with seven well-established classifiers and comprehensive evaluation metrics. It is worth noting that Kuo et al. originally introduced this dataset as a data descriptor, demonstrating standardized collection and technical validation of clinical breathomics data but without performing predictive modeling or biomarker interpretation^20^. Our study extends their contribution by establishing baseline classification performance and presenting interpretable analyses that highlight disease-related VOCs. This distinction emphasizes the complementary nature of our work in advancing the clinical utility of breathomics data.

Several prior studies in the breathomics domain have explored the utility of exhaled VOCs for respiratory disease classification, yet most remain limited in scope or methodology. For instance, Tian et al. conducted a cross-sectional study using a portable micro-GC system to differentiate among COPD, asthma, PRISm, and healthy individuals based on exhaled breath profiles^36^. While their approach successfully identified disease-specific VOC panels and developed several classification models including random forest and SVM, they did not adopt model interpretability tools such as SHAP to explain feature contributions. Moreover, their focus was largely on binary or pairwise disease discrimination rather than full multi-class classification. Similarly, studies based on the National Health and Nutrition Examination Survey (NHANES) dataset, such as those by Liu et al., examined the relationship between VOC metabolites (measured in blood or urinary) and COPD risk^18^. These works provided valuable epidemiological insights but did not utilize breath-based VOCs or machine learning models with interpretability. Furthermore, statistical models like logistic regression were primarily used without stratified or cross-validation strategies. More broadly, many existing studies employed binary classification designs such as differentiating COPD from healthy controls or tuberculosis from non-TB cases using VOCs extracted through various analytical platforms. For example, Fu et al. achieved high accuracy in detecting pulmonary tuberculosis from exhaled breath using HPPI-TOF-MS and ensemble classifiers like XGBoost^34^. However, their setting involved binary discrimination and lacked interpretability tools.

In contrast, our study directly addresses these limitations. First, we deal with a clinically relevant multi-class classification problem involving three major respiratory diseases: asthma, bronchiectasis, and COPD. Second, we employ a diverse set of machine learning models with robust evaluation techniques, specifically using 5-fold nested cross-validation combined with bootstrap confidence intervals, ensuring unbiased performance estimation and quantification of uncertainty. Lastly and most critically, we apply SHAP-based interpretability techniques to reveal key disease-specific biomarkers, enabling biological transparency and clinical interpretability.

Despite these encouraging findings, there are several limitations to consider. First, the dataset comprises only 121 patient samples across three disease classes, which limits the statistical power and generalizability of the findings. Although nested cross-validation with outer test folds and bootstrapping provided robust within-dataset evaluation—demonstrating stability through small variability across folds (Table 1) and quantifying uncertainty via confidence intervals (Table S1)—true external generalizability can only be established through validation on independent and larger cohorts. A further limitation is that we restricted the analysis to VOCs commonly detected across all three disease groups to maintain fairness and comparability across models. While this avoided systematic missingness that classical algorithms cannot handle, it also meant that potentially informative disease -specific VOCs were not considered in the current analysis. Second, the dataset lacks critical metadata such as age, sex, medication history, comorbidities, and smoking status. These variables are known to influence the composition of exhaled VOCs and may act as confounding factors in disease classification. Previous studies have demonstrated that smoking can significantly alter the exhaled metabolome, with specific compounds elevated or suppressed due to tobacco exposure^42^. On the other hand, medications such as corticosteroids have been shown to modulate VOC profiles in asthmatic patients^43^. Without the ability to adjust for these covariates, the observed VOC signatures may partially reflect non-disease-related factors, potentially biasing the model’s interpretation. Third, while SHAP analysis offers valuable insights into the contribution of individual features to model predictions, it remains a correlational tool rather than a causal inference framework. SHAP identifies VOCs that are statistically associated with disease classifications, but it does not confirm whether these metabolites play a mechanistic role in pathogenesis. Consequently, the VOCs identified by SHAP analysis should be interpreted as putative biomarkers and further validated in future studies. Another limitation concerns the chemical annotations of specific VOCs. Some SHAP-identified compounds, such as siloxane derivatives (e.g., CID 7874) and aromatic hydrocarbons (e.g., CID 9231) have been reported in the GC-MS literature as potential column bleed artifacts or xenobiotic contaminants rather than endogenous metabolites. The dataset we used inherited its compound identifications directly from Kuo et al., but mass spectral match scores or validation metrics were not provided, making it difficult to fully assess the confidence of these annotations. Therefore, while our interpretability analysis demonstrates statistically influential features, these should be viewed as provisional markers requiring careful biochemical validation to exclude analytical artifacts or environmental sources.

Several SHAP-identified VOCs were associated with processes like oxidative stress and chronic airway inflammation. However, clarifying their detailed roles in disease progression, including signaling pathways, protein-protein interactions, or transcriptional regulation, is beyond the scope of this study. Breathomics data alone cannot resolve causal mechanisms, and such insights would require integration with transcriptomic, proteomic, or experimental validation approaches. Future work should therefore aim to combine VOC-based signatures with multi-omics datasets and biological assays to clarify the regulatory pathways through which these metabolites influence respiratory disease pathogenesis.

Additionally, while our use of nested cross-validation mitigates overfitting risk and provides robust performance estimates, the reported metrics should still be interpreted cautiously. Reporting confidence intervals across outer folds helps quantify variability, but optimism bias may persist without external validation. Thus, independent cohorts remain essential for fully confirming generalizability.

Beyond these limitations, we acknowledge that our study did not include deep learning approaches such as convolutional neural networks (CNNs) or transformer-based models. This decision was motivated by the relatively small cohort size, which increases the risk of overfitting with high-capacity models, and by our methodological focus on interpretability using established classical machine learning algorithms^44^. Deep learning models may indeed capture more complex feature interactions in larger datasets, but their interpretability remains challenging compared to SHAP-enabled classical models^45^. Future studies with expanded cohorts could therefore explore deep learning architectures in parallel, to assess whether their predictive advantages can be realized while maintaining clinical transparency.

This study contributes a quantitatively strong, interpretable, and reproducible machine learning framework for VOC-based classification of respiratory diseases. Our results demonstrate that ensemble models like XGBoost combined with SHAP analysis not only achieve high classification performance but also provide biologically plausible insights into disease-specific VOC signatures. This combination of performance and interpretability enhances the clinical translational potential of breathomics-based diagnostics. Future work should aim to validate these findings in independent clinical settings, incorporate additional clinical covariates, and explore multi-modal integration with other omics data to further improve diagnostic accuracy and robustness.

## Conclusion

We present a reproducible, interpretable machine learning baseline for classifying asthma, bronchiectasis, and COPD from a public GC-MS dataset of exhaled breath VOCs. Across seven supervised classifiers, particularly ensemble-based models such as XGBoost, we observed strong diagnostic performance across multiple evaluation metrics. The incorporation of SHAP-based interpretability further enabled the identification of key VOCs driving disease-specific predictions, providing candidate biochemical markers and reinforcing the biological plausibility of model outputs. These results underscore the utility of breathomics not only as a practical diagnostic alternative to invasive or subjective diagnostic tools but also as a pathway toward transparent and explainable respiratory disease classification. By establishing this reproducible and interpretable baseline, our work provides a reference point for future methodological advancements and external validation in breathomics-based diagnostics.

## Materials and methods

### Dataset description & data preprocessing

This study utilized a publicly available clinical breathomics dataset published by Kuo et al., which comprises GC-MS profiles of exhaled breath from individuals with three common respiratory diseases: asthma, bronchiectasis, and COPD^20^. All samples were collected in a clinical setting as part of the cohort reported by Kuo et al., where exhaled breath condensates were analyzed using GC-MS and annotated with PubChem Compound IDs (CIDs) and its corresponding IUPAC names^20^. The dataset was specifically curated to support the development of machine learning algorithms for non-invasive respiratory disease classification based on VOCs.

The dataset comprises 53 samples from patients with asthma, 35 samples from individuals with bronchiectasis, and 33 samples from those with COPD. For each disease group, GC-MS was used to identify and quantify the chemical components present in exhaled breath condensate samples. The resulting VOC peak tables provide metabolite intensity values for a varying number of compounds per disease: 130 for asthma, 119 for bronchiectasis, and 122 for COPD.

Because these compound lists were not identical, direct concatenation across groups would have resulted in systematic missingness whenever a VOC was absent in one group but present in another. To enable fair and consistent comparisons across disease groups, only the metabolites commonly detected in all three diseases were selected yielding 78 shared VOCs. This decision was also methodological: if a VOC appeared uniquely in one disease group, its values would be missing in the others, which cannot be handled consistently by most classical machine learning algorithms (e.g., kNN, SVM, logistic regression). Although algorithms such as XGBoost can accommodate missing values internally, restricting the feature set to shared VOCs ensured comparability across all classifiers and avoided introducing artificial missingness. However, two features in the COPD dataset (CID: 17835 and 622436) were found to be duplicated with substantial differences in values. To mitigate potential bias, these duplicated features were removed, resulting in a final set of 76 shared VOCs, which was used as the feature set for subsequent machine learning classification. The complete list of these VOCs with their CID numbers and IUPAC names is provided in Supplementary Table S2.

After aligning the datasets based on the 76 common VOCs, a unified dataset was formed with 121 total samples and 76 shared features. To ensure feature comparability, VOC intensity values were standardized using z-score normalization (zero mean and unit variance). Importantly, normalization parameters (mean and standard deviation) were always fitted on training folds within cross-validation and then applied to the corresponding validation folds, thereby preventing information leakage.

### Classification models

To classify respiratory diseases based on breath-derived VOC features, we implemented and evaluated seven representative supervised machine learning algorithms: kNN, logistic regression, naïve Bayes, decision tree, SVM, random forest, and XGBoost. These models were selected to represent a broad spectrum of classification paradigms in supervised learning, from probabilistic and geometric approaches to ensemble-based methods. Each model has distinct strengths that render it suitable for the high-dimensional, moderately sized clinical breathomics dataset used in this study.

KNN is a simple yet effective instance-based learning algorithm that classifies a sample based on the majority label among its nearest neighbors in feature space^46^. It is particularly suitable for limited sample sizes and does not rely on parametric assumptions, making it appropriate for exploratory classification tasks.

Logistic regression is a classical linear model and widely used for classification tasks due to its interpretability and computational efficiency^47^. Although it assumes linear relationships between features and the log-odds of the outcome, it provides high interpretability and serves as a robust baseline in many classification problems.

Naïve Bayes is a probabilistic classifier based on Bayes’ theorem with the assumption of feature independence^48^. Despite its simplicity, naïve Bayes is remarkably effective for high-dimensional settings, even when its underlying assumptions are violated.

Decision tree is a non-parametric model that splits the feature space recursively based on optimal thresholds, yielding interpretable tree structures^49^. Decision tree can capture nonlinear relationships and feature interactions between variables, which is advantageous in analyzing complex biological signals such as VOC profiles.

SVM is a margin-based classifier that constructs an optimal hyperplane to separate classes, and they are particularly effective in high-dimensional spaces^50^. By employing kernel functions, SVM can model complex nonlinear decision boundaries, making them suitable for VOC-based classification where interactions among metabolites may be intricate.

Random forest is an ensemble learning method that builds multiple decision trees using bootstrapped datasets and random subsets of features^51^. It offers high classification accuracy and robustness against overfitting while also enabling feature importance estimation.

XGBoost is a state-of-the-art implementation of gradient boosting decision trees^38^. It builds trees sequentially, optimizing a regularized objective function, and offers superior performance on noisy and heterogeneous datasets, such as those encountered in clinical VOC datasets.

To obtain unbiased estimates of generalization performance, we employed a 5-fold nested cross-validation framework. In this setup, the outer folds provided strictly held-out test sets for model evaluation, while the inner folds were used exclusively for hyperparameter tuning via grid search. The specific hyperparameter grids searched for each classifier are summarized in Table 4. Hyperparameters were chosen to maximize the macro-averaged F1-score, which balances performance across all three disease classes despite class imbalance.

Classification performance was assessed using accuracy, AUC, precision, sensitivity, and F1-score. Given the multi-class nature of the task, we reported macro-averaged metrics to equally weight all classes regardless of sample size. For ROC analysis, individual class-wise ROC curves were generated using the one-vs-rest strategy, and a macro-average ROC curve was constructed by averaging true positive rates (TPRs) at common false positive rate (FPR) thresholds. The area under this macro-average ROC curve (macro-AUC) was then reported as a class-balanced measure of overall discrimination performance. This nested cross-validation framework provides a rigorous and unbiased estimate of model generalization performance.

**Table 4:** Hyperparameter search spaces for each classification model used in GridSearchCV. (Abbreviations: kNN = k-nearest neighbors, LR = logistic regression, NB = naïve Bayes, DT = decision tree, SVM = support vector machine, RF = random forest, and XGBoost = extreme gradient boosting. Default parameters indicates models without tunable hyperparameters.).

Models	Scikit-learn parameter names	Hyperparameter grid
kNN	n_neighbors	[3, 5, 7, 9, 11, 13, 15]
LR	C	[0.001, 0.01, 0.1, 1, 10, 100]
NB	–	Default parameters (GaussianNB)
DT	max_depth	[5, 10, 15, 20, None]
	min_samples_split	[2, 5, 10, 15]
SVM	C	[0.01, 0.1, 1, 10, 100]
	kernel	[‘linear’, ‘rbf’, ‘poly’]
	degree	[2, 3, 4, 5]
RF	n_estimators	[50, 100, 200, 500]
	max_depth	[5, 10, 15, 20, None]
	min_samples_split	[2, 5, 10, 15]
XGBoost	n_estimators	[50, 100, 200, 500]
	max_depth	[3, 6, 9]
	learning_rate	[0.01, 0.1, 0.2]
	subsample	[0.6, 0.8, 1.0]
	colsample_bytree	[0.6, 0.8, 1.0]

To further quantify the stability of these results, we performed nonparametric bootstrapping (1,000 resamples) on the final refitted models. This analysis yielded 95% confidence intervals for all evaluation metrics reported in Table S1, complementing the nested cross-validation results and providing an additional assessment of robustness.

**Fig. 4:**
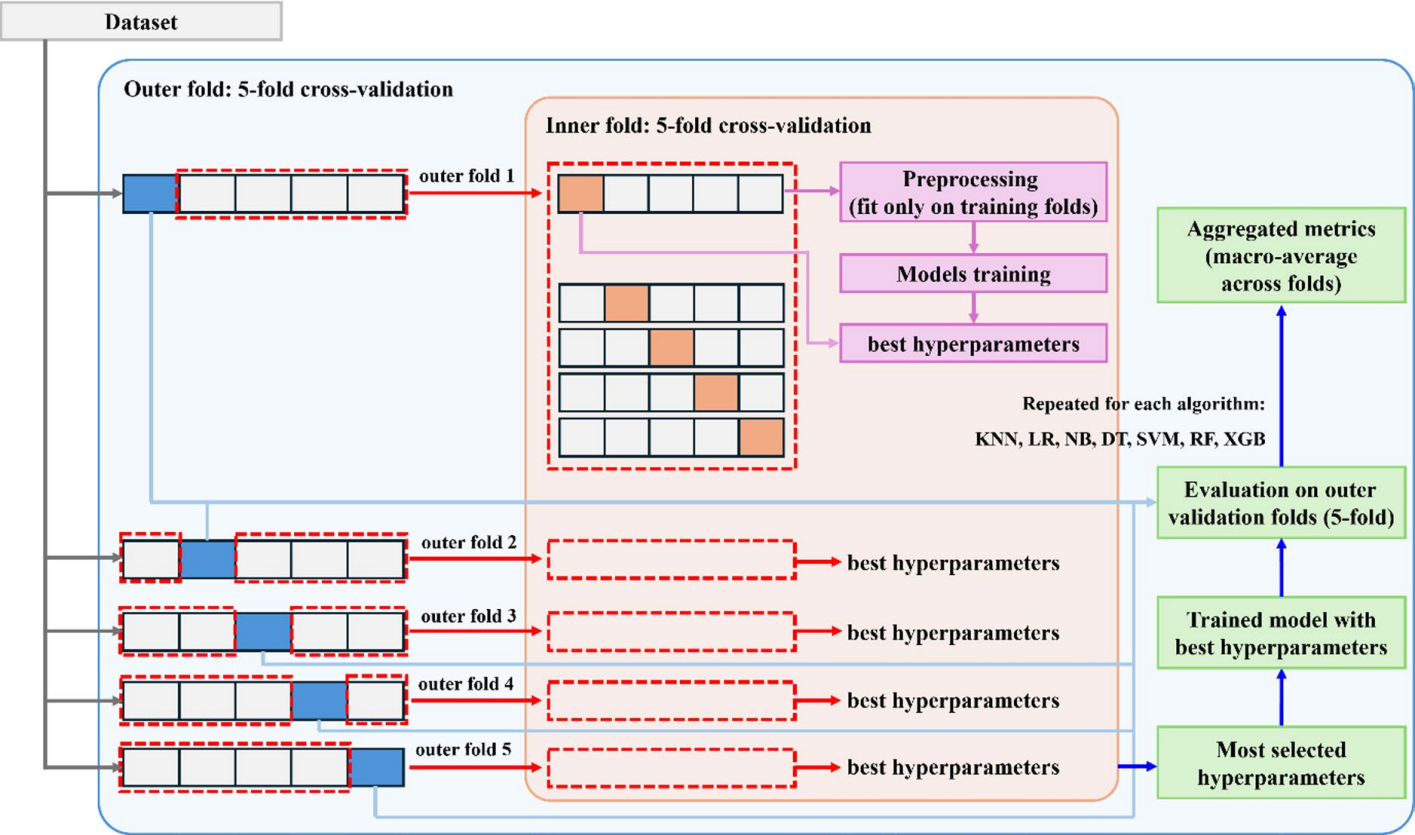
Schematic flowchart of the experimental design. The dataset was split into 5 outer folds for unbiased model evaluation, with inner 5-fold cross-validation used for hyperparameter tuning and preprocessing applied only to training folds.

To enhance transparency of the experimental design, Fig. 4 provides a schematic overview of the nested cross-validation pipeline used in this study. The flowchart illustrates how the dataset was partitioned into outer folds for unbiased performance evaluation and inner folds for hyperparameter tuning, with preprocessing (z-score normalization) applied only within the training partitions to prevent data leakage. Aggregated metrics were computed across the outer folds, while the most frequently selected hyperparameters were used to retrain the final models for SHAP-based interpretability.

### Model interpretation

To interpret the trained classification models and identify key VOC features associated with respiratory disease, we employed SHAP on the final refitted XGBoost model to assign importance values based on each feature’s contribution to model predictions. SHAP provides both global interpretation that summarizes feature impact across the entire dataset and local interpretation that explains individual predictions at the sample level.

In the context of multi-class classification, SHAP estimates class-wise contributions by measuring how each feature influences the predicted probability of a specific class relative to a baseline (i.e., the expected value). These SHAP values are computed by evaluating all possible feature subsets of features and calculating the marginal contribution of each feature. This additive property ensures both consistency and local accuracy in model explanation.

For global interpretability, we computed the mean absolute SHAP value of each VOC across all samples and classes using the XGBoost model. This metric provides a quantitative estimate of the average magnitude by which each feature influences the model’s decision, allowing for a ranked comparison of VOC importance. Furthermore, we decomposed these global effects into class-specific SHAP contributions which represent the VOCs most influential for differentiating each individual disease class, asthma, bronchiectasis, COPD. This is particularly valuable in multi-class settings, where distinct VOCs may be relevant for different diagnostic boundaries.

Beyond global importance, SHAP also supports local interpretability by quantifying how individual features push model predictions toward or away from specific class probabilities. These local explanations are essential for understanding cases with borderline predictions or misclassifications, thereby supporting trust and transparency in machine learning-assisted diagnosis.

By applying SHAP to the XGBoost model, we aimed to uncover the most influential VOCs that drive the differentiation among asthma, bronchiectasis, and COPD. This analysis pipeline not only improves model interpretability but also provides a biologically plausible means of identifying candidate breath biomarkers associated with disease-specific signatures.

### Implementation details

All analyses were conducted in Python (version 3.12.7). Model training and evaluation were implemented using scikit-learning (version 1.5.1) for logistic regression, kNN, naïve Bayes, decision tree, SVM, and random forest. XGBoost models were trained with the XGBoost package (version 3.0.2). Data preprocessing and handling were performed with numpy (version 1.26.4) and pandas (version 2.2.3). Visualization of ROC curves and confusion matrices was carried out using matplotlib (version 3.10.0). SHAP-based interpretability analyses were performed with the shap package (version 0.48.0). To ensure reproducibility, the full source code used for data preprocessing, model training, and SHAP-based interpretation is publicly available at https://github.com/danniskang/breathomics. These package versions and the accompanying code repository reflect the exact computational environment used in this study, ensuring reproducibility of the reported results.

## Supplementary Information

Below is the link to the electronic supplementary material.

Supplementary Material 1

## Data Availability

The dataset for this study is publicly accessible at [https://doi.org/10.6084/m9.figshare.23522490.v6](https:/doi.org/10.6084/m9.figshare.23522490.v6) and its accessibility was verified on July 31, 2025.
