# Supplementary material for: A baseline study of interpretable machine learning using GC-MS breath VOCs for classifying asthma, bronchiectasis, and COPD
Source: Sci Rep. 2025 Dec 23;15:44392. doi: 10.1038/s41598-025-28143-x (PMC12728165; doi:10.1038/s41598-025-28143-x)
Supplement: Supplementary file 1 — Supplementary Material 1 [file 41598_2025_28143_MOESM1_ESM.docx]

**A Baseline Study of Interpretable Machine Learning Using GC-MS Breath VOCs for Classifying Asthma, Bronchiectasis, and COPD**

Eun-Ji Ko^†^, Si-On Bae^†^ and Daesung Kang*

School of Bio-Health Convergence, College of Natural Sciences, Sungshin Women’s University Woonjung Green Campus, Seoul, Republic of Korea

Figure S1. Class-wise ROC curves for seven classification models using the one-vs-rest (OvR) strategy. Each panel shows ther per-class ROC curves (asthma, bronchiectasis, COPD) together with the macro-average ROC curve obtained from outer cross-validation. (kNN: k-nearest neighbors, LR: logistic regression, NB: naïve Bayes, DT: decision tree, SVM: support vector machine, RF: random forest, XGBoost: extreme gradient boosting.


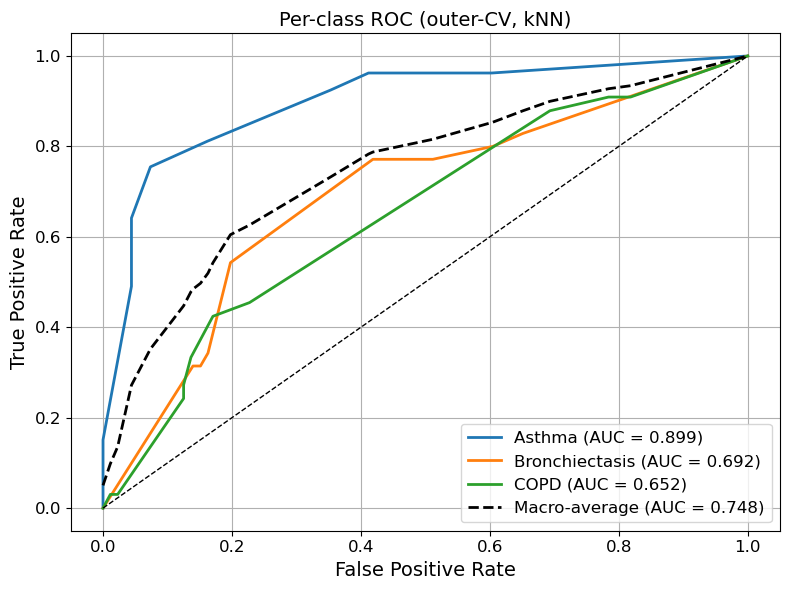

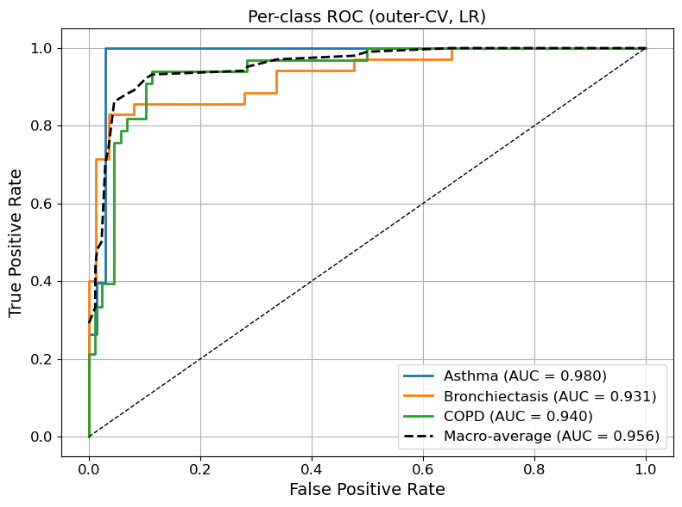


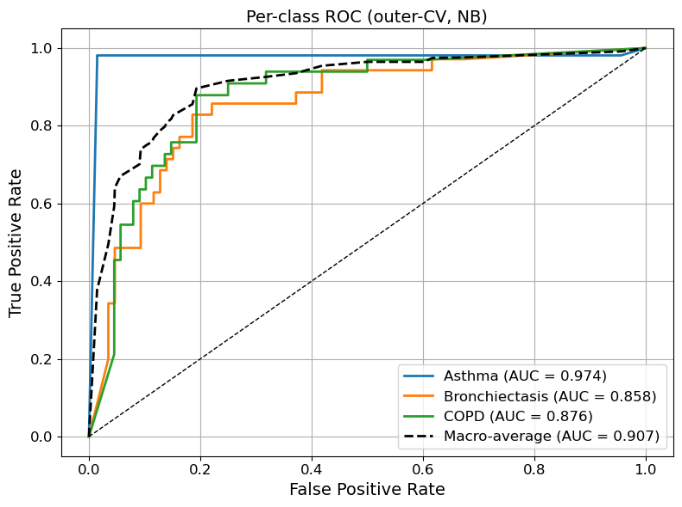

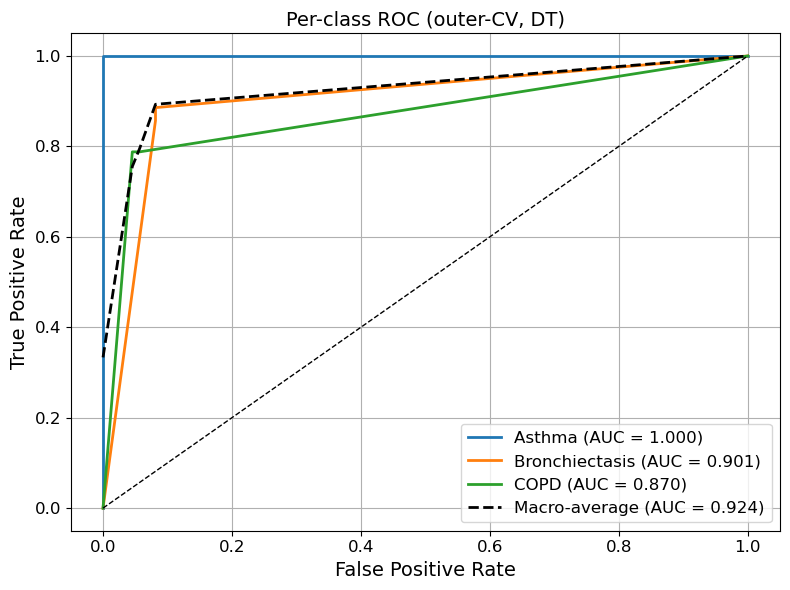


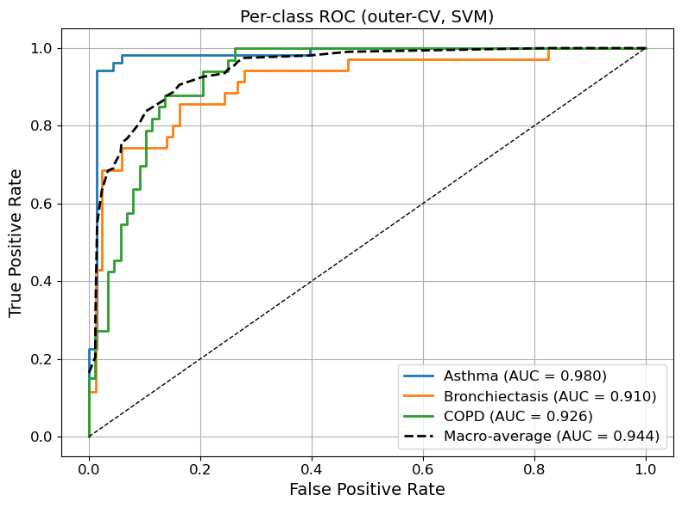

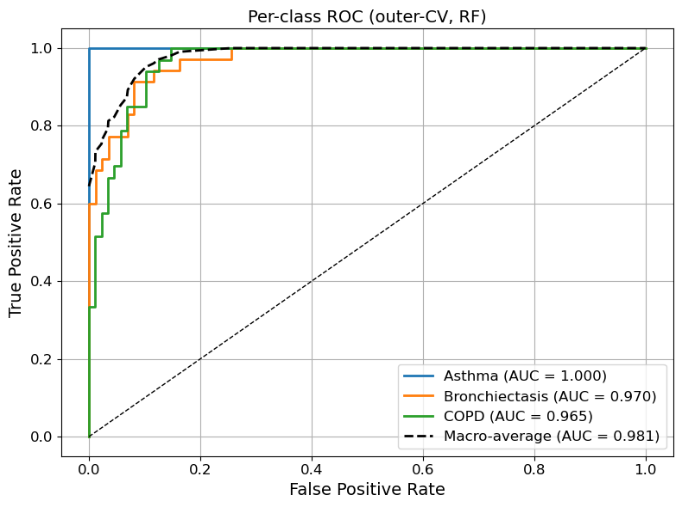


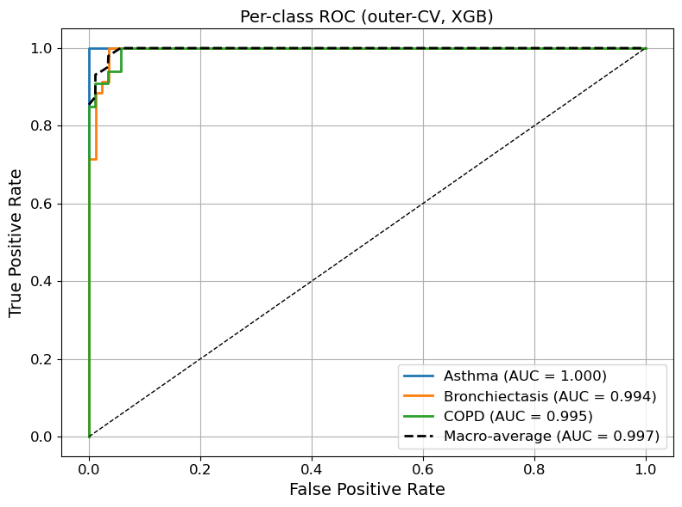


Figure S2. Class-wise SHAP feature importance plots for all 76 shared VOCs. Each bar chart presents the mean absolute SHAP values of all compounds, calculated separately for asthma, bronchiectasis, and COPD, based on the refitted XGBoost model using the best hyperparameters from nested cross-validation. VOCs are identified by their PubChem CID numbers on the y-axis, and bar lengths represent the average contribution of each VOC to the corresponding class prediction.


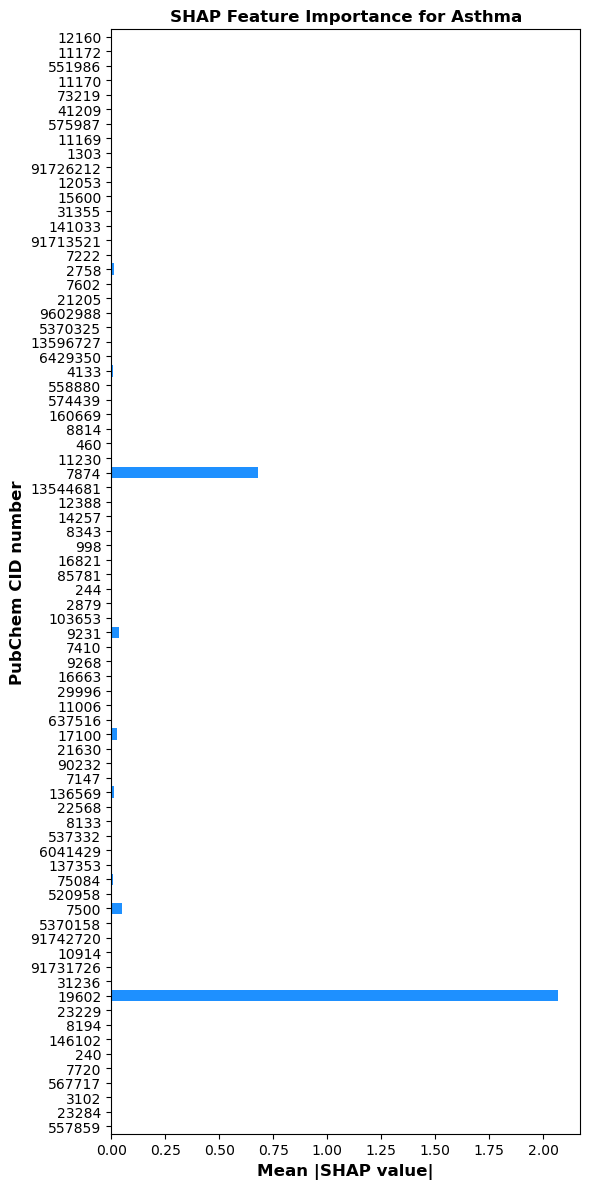

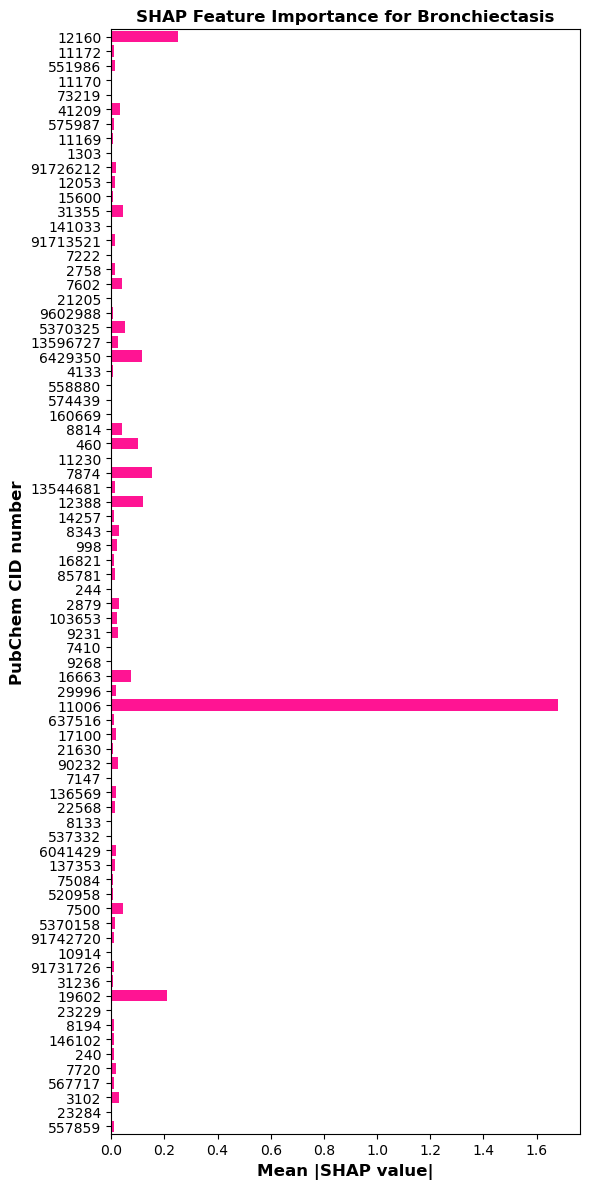


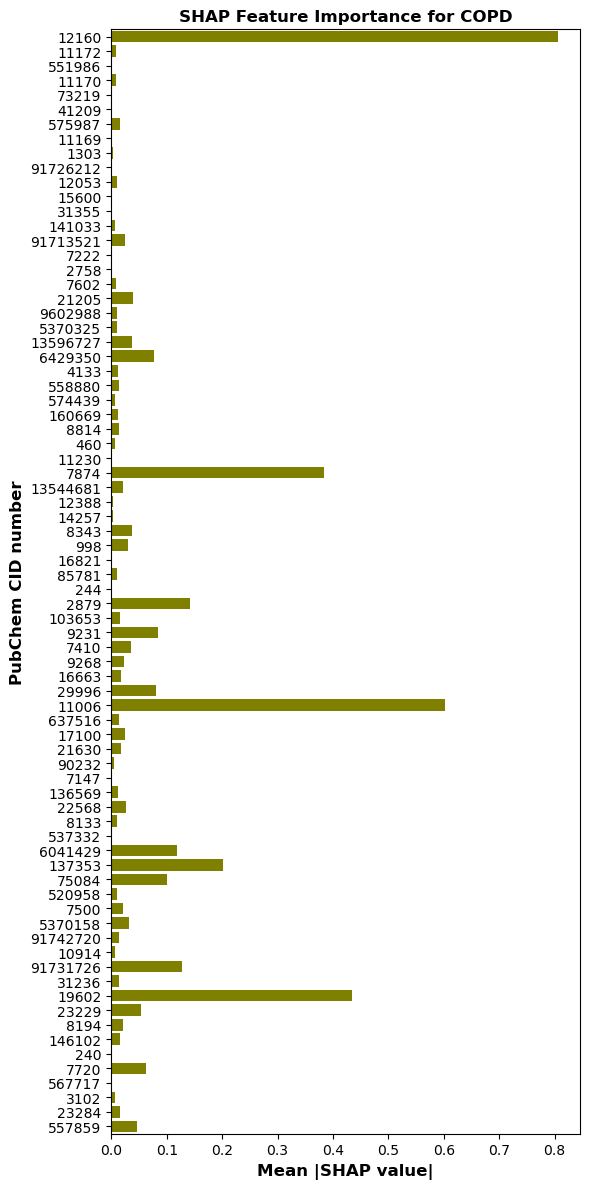


Figure S3. SHAP summary plot showing the top 10 VOCs contributing to classification across asthma, bronchiectasis, and COPD. Each horizontal bar represents a VOC, identified by its IUPAC names on the y-axis, with bar length and color indicating the magnitude and class-specific contribution.


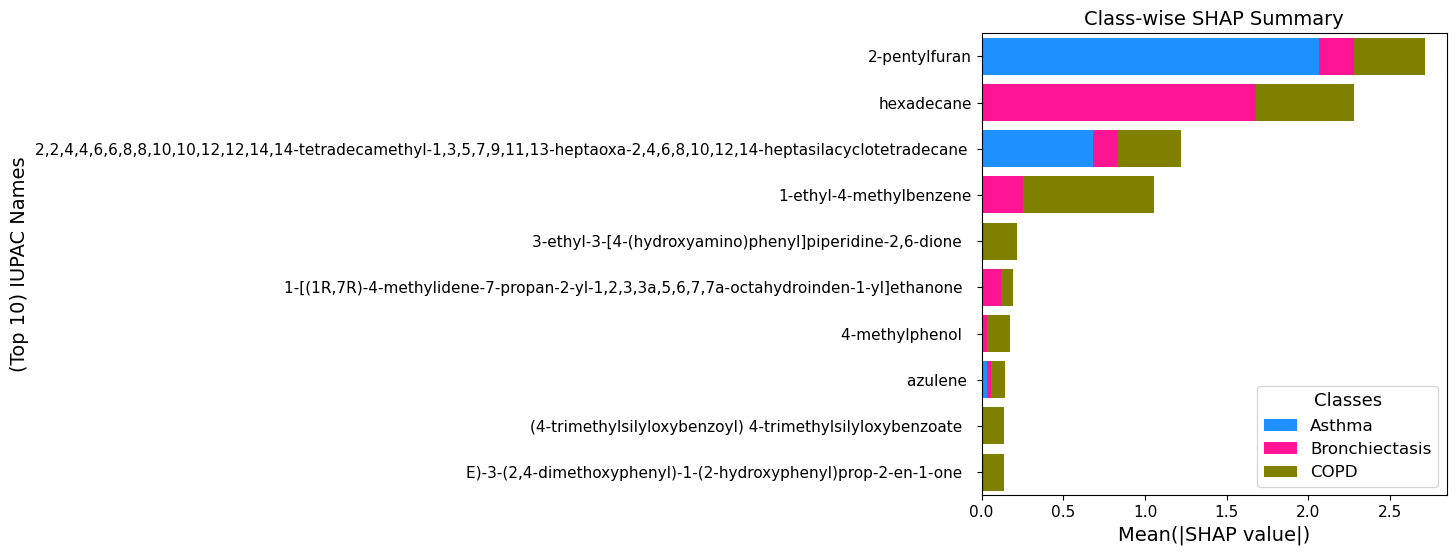


Figure S4. Class-wise SHAP feature importance plots for all 76 shared VOCs. Each bar chart presents the mean absolute SHAP values of all compounds, calculated separately for asthma, bronchiectasis, and COPD, based on the refitted XGBoost model using the best hyperparameters from nested cross-validation. VOCs are identified by their IUPAC names on the y-axis, and bar lengths represent the average contribution of each VOC to the corresponding class prediction.


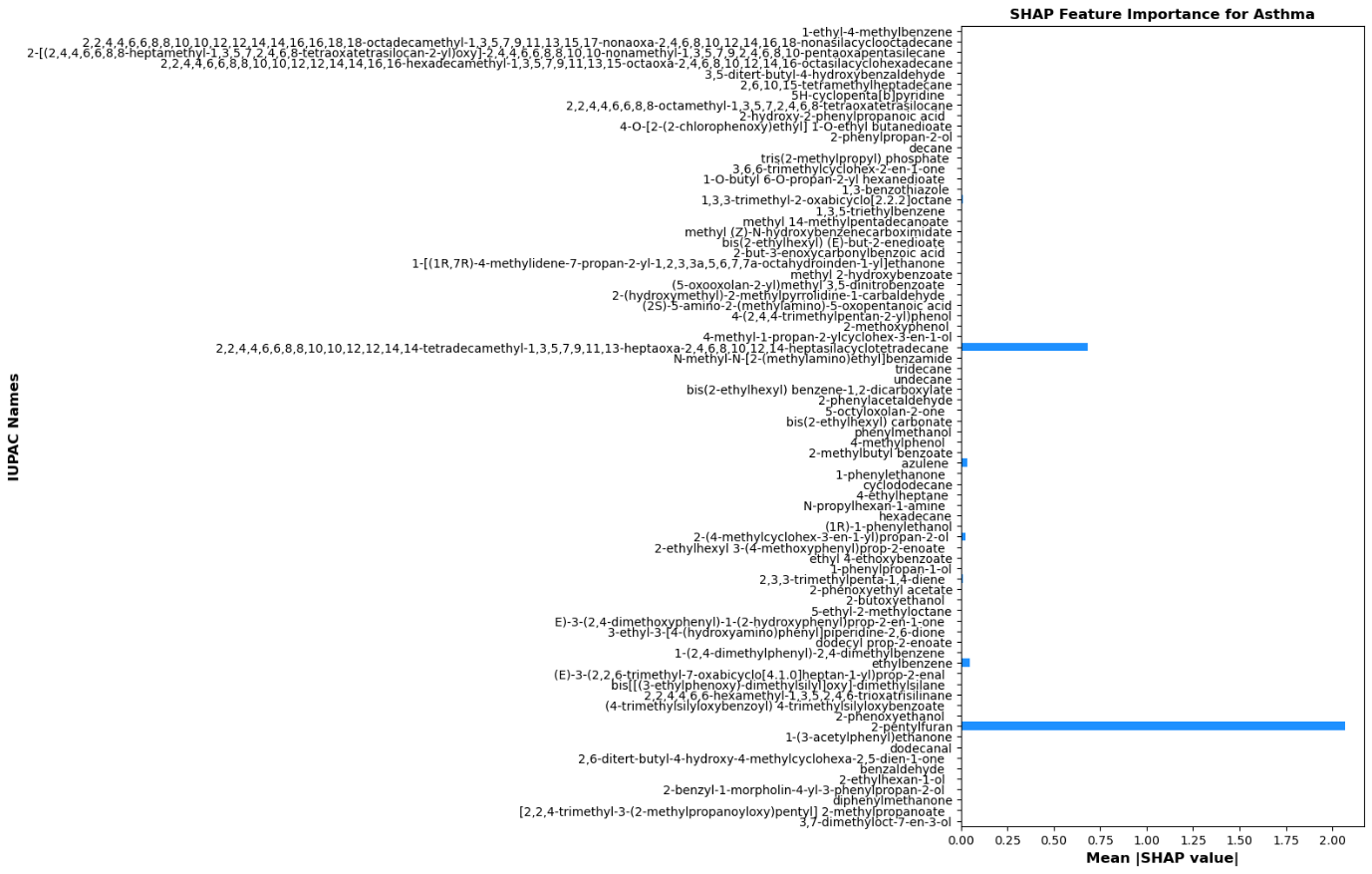


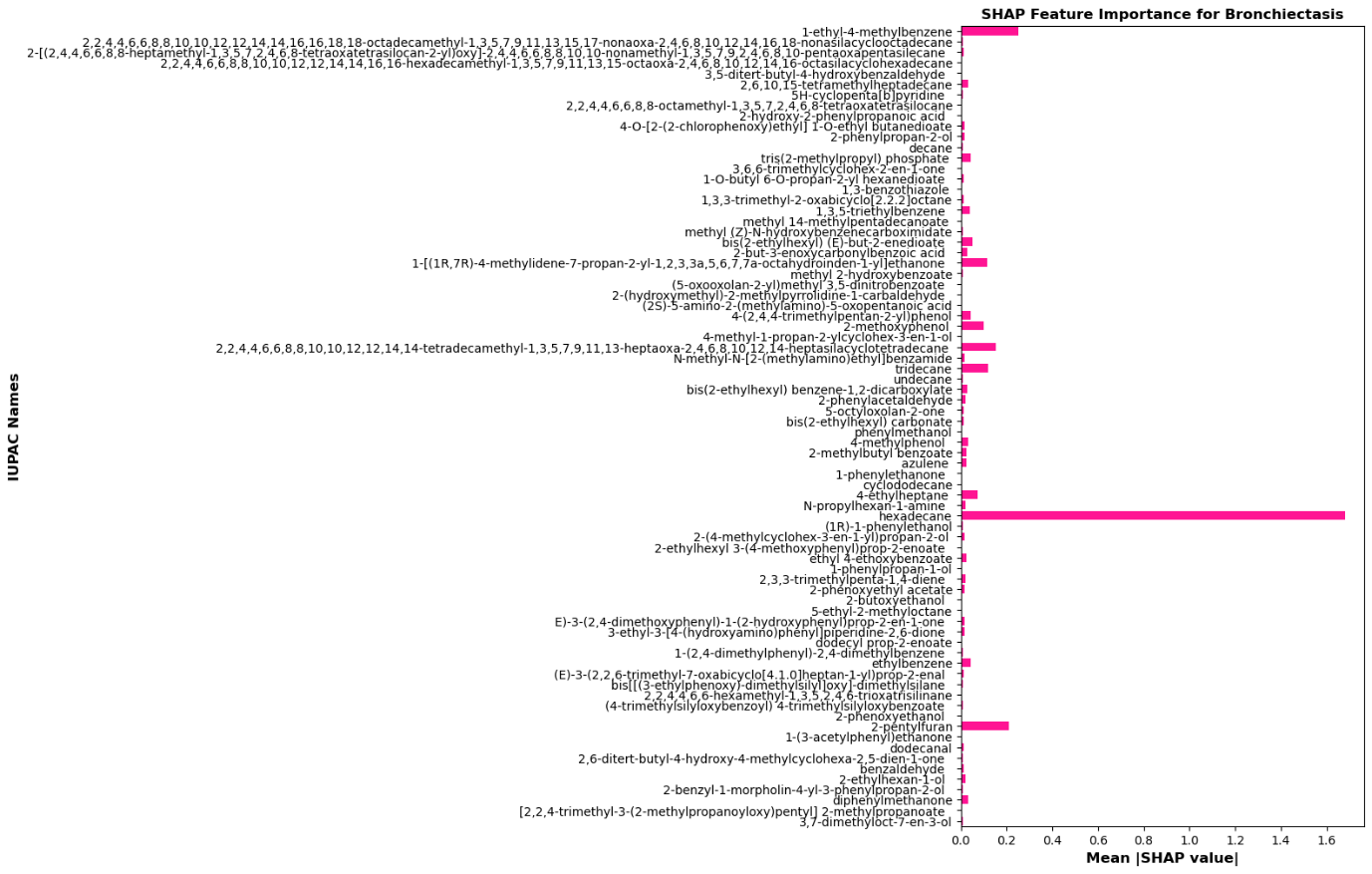


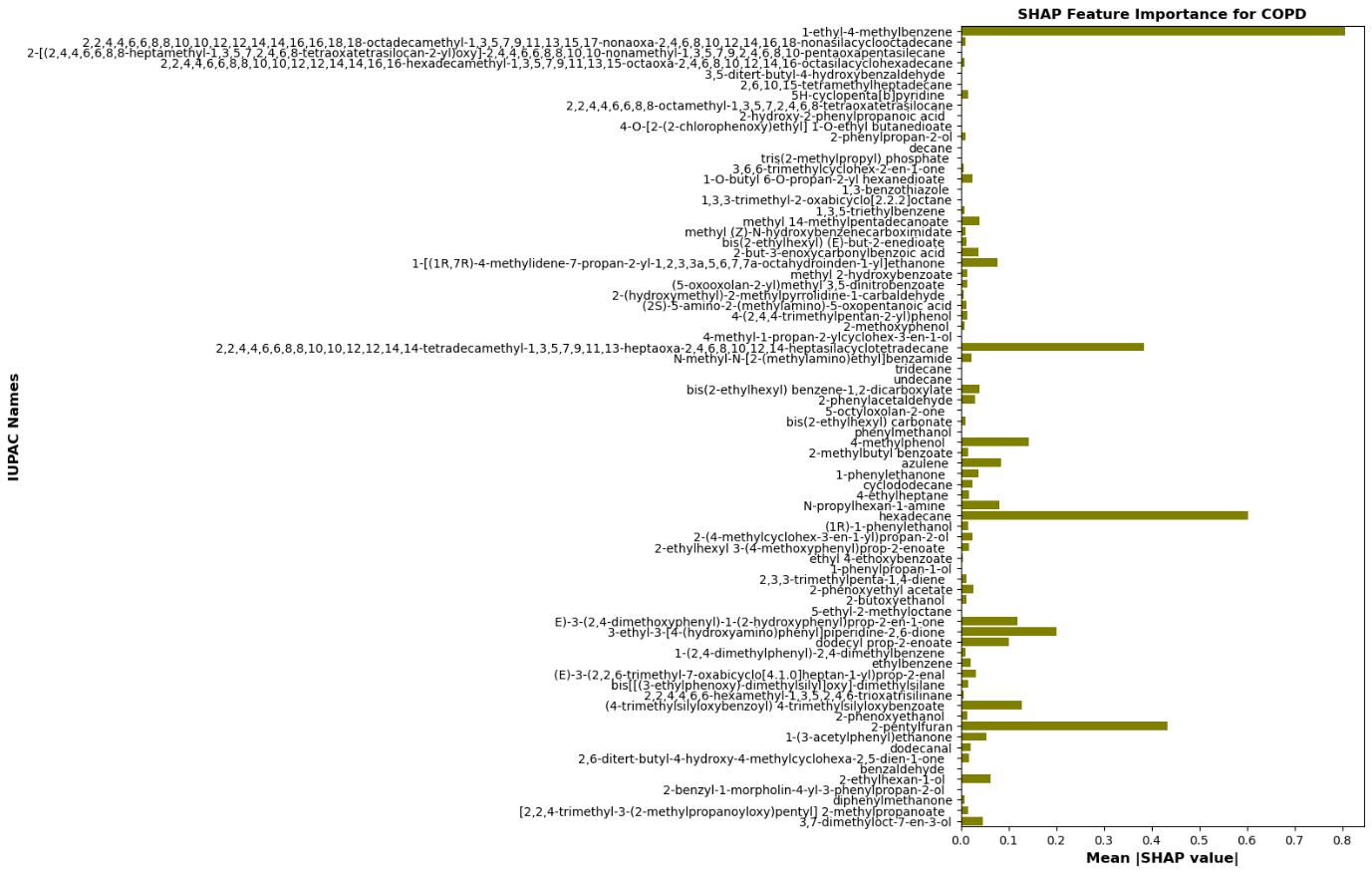


Figure S5. Class-wise SHAP beeswarm plots for asthma, bronchiectasis, and COPD. Each plot displays the 9 most influential VOCs for that disease, along with the summed contribution of all remaining 67 VOCs. VOCs are identified by their IUPAC names on the y-axis. Each point corresponds to one patient sample, with horizontal position indicating the SHAP value (impact on model output). Positive SHAP values indicate that a VOC increases the likelihood of the disease prediction, whereas negative values decrease it. Color represents the relative feature value for that sample (red = high, blue = low).


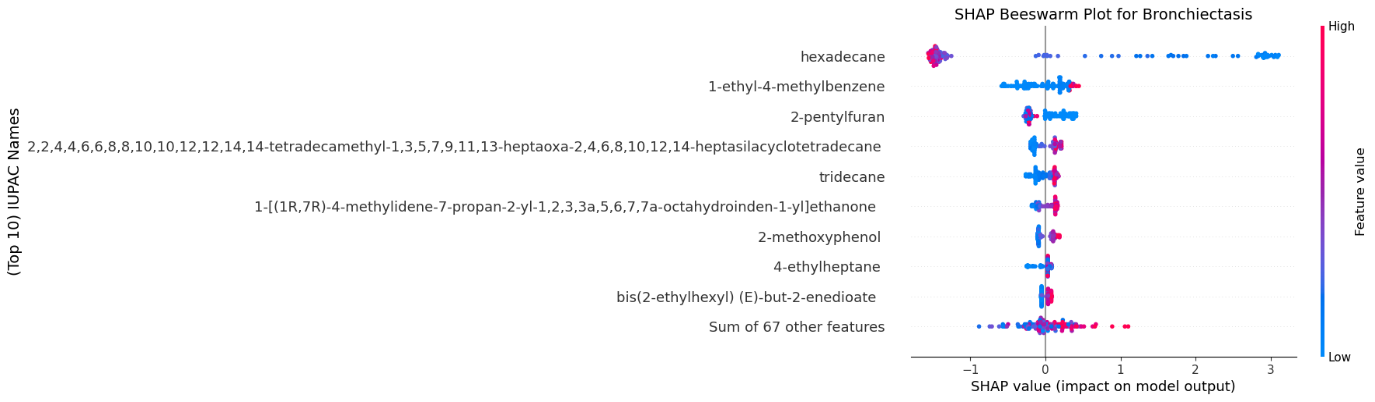


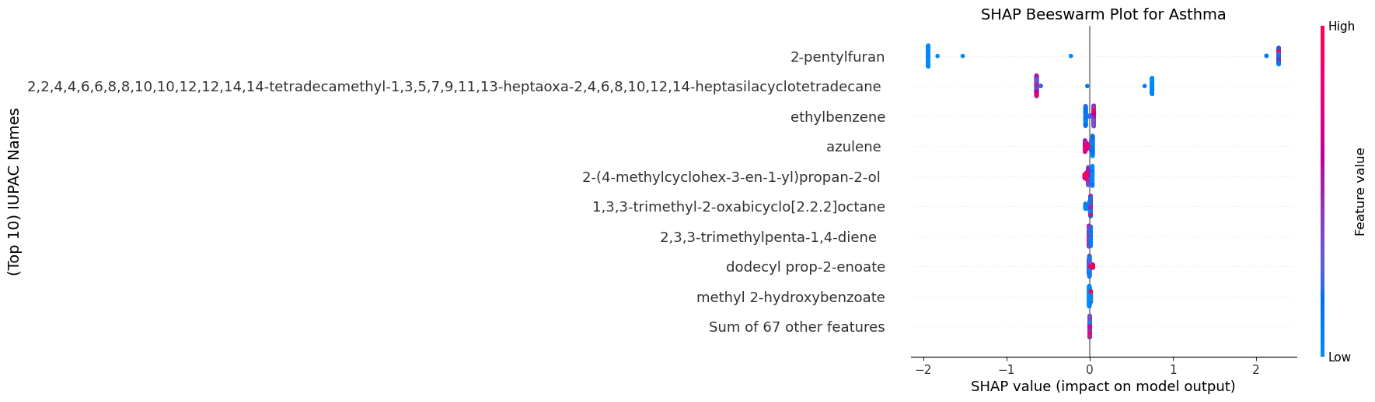


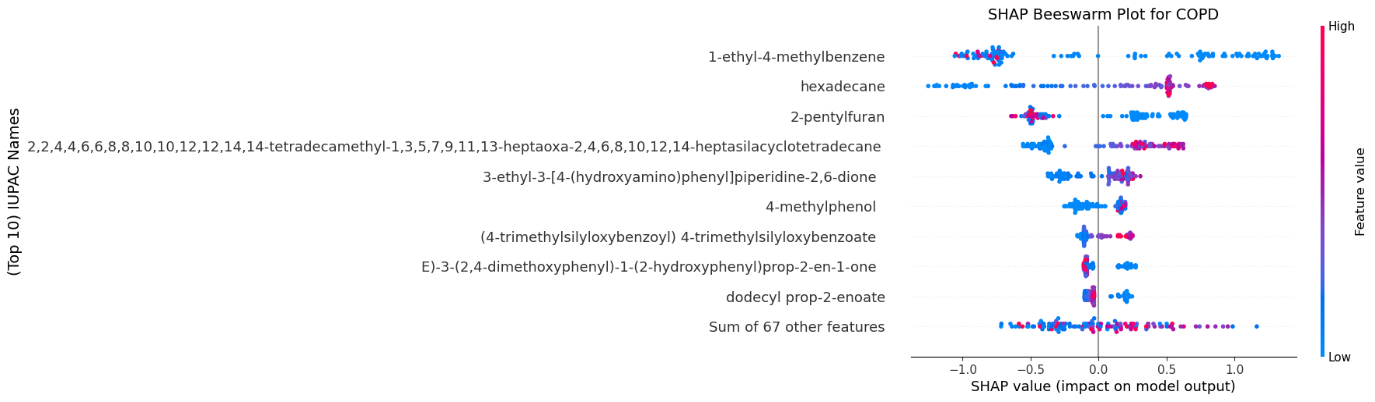


Table S1. Bootstrap-estimated classification performance with 95% confidence intervals for seven machine learning models. (Values are presented as mean estimates followed by the 95% confidence interval in parentheses, obtained from 1,000 bootstrap resamples of the refitted model predictions.)

| **Models** | **Accuracy (%)** | **AUC** | **Precision** | **Sensitivity** | **F1-score** |
| --- | --- | --- | --- | --- | --- |
| KNN | 56.46  (40.91 ~ 70.27) | 0.742  (0.630 ~ 0.842) | 0.547  (0.391 ~ 0.695) | 0.541  (0.390 ~ 0.677) | 0.526  (0.375 ~ 0.665) |
| LR | 83.01  (70.72 ~ 93.33) | 0.930  (0.858 ~ 0.989) | 0.815  (0.687 ~ 0.926) | 0.812  (0.683 ~ 0.929) | 0.805  (0.671 ~ 0.922) |
| NB | 78.29  (66.67 ~ 88.89) | 0.896  (0.817 ~ 0.960) | 0.758  (0.626 ~ 0.879) | 0.750  (0.638 ~ 0.871) | 0.738  (0.611 ~ 0.860) |
| DT | 89.69  (79.55 ~ 97.87) | 0.916  (0.836 ~ 0.984) | 0.883  (0.765 ~ 0.976) | 0.880  (0.765 ~ 0.978) | 0.875  (0.761 ~ 0.975) |
| SVM | 80.56  (67.33 ~ 91.49) | 0.923  (0.841 ~ 0.983) | 0.789  (0.651 ~ 0.909) | 0.784  (0.644 ~ 0.901) | 0.777  (0.639 ~ 0.899) |
| RF | 90.54  (80.00 ~ 97.92) | 0.979  (0.931 ~ 0.999) | 0.896  (0.787 ~ 0.978) | 0.893  (0.778 ~ 0.978) | 0.886  (0.766 ~ 0.975) |
| XGBoost | 93.06  (83.99 ~ 100.00) | 0.991  (0.967 ~ 1.000) | 0.924  (0.833 ~ 1.000) | 0.921  (0.823 ~ 1.000) | 0.917  (0.813 ~ 1.000) |

Table S2. The common PubChem CID and IUPAC Name lists for each disease used in this study are provided below.

| PubChem CID | IUPAC Name |
| --- | --- |
| 17100 | 2-(4-methylcyclohex-3-en-1-yl)propan-2-ol |
| 6429350 | 1-[(1R,7R)-4-methylidene-7-propan-2-yl-1,2,3,3a,5,6,7,7a-octahydroinden-1-yl]ethanone |
| 16821 | 5-octyloxolan-2-one |
| 136569 | 2,3,3-trimethylpenta-1,4-diene |
| 146102 | 2,6-ditert-butyl-4-hydroxy-4-methylcyclohexa-2,5-dien-1-one |
| 29996 | N-propylhexan-1-amine |
| 7720 | 2-ethylhexan-1-ol |
| 13596727 | 2-but-3-enoxycarbonylbenzoic acid |
| 551986 | 2-[(2,4,4,6,6,8,8-heptamethyl-1,3,5,7,2,4,6,8-tetraoxatetrasilocan-2-yl)oxy]-2,4,4,6,6,8,8,10,10-nonamethyl-1,3,5,7,9,2,4,6,8,10-pentaoxapentasilecane |
| 23284 | [2,2,4-trimethyl-3-(2-methylpropanoyloxy)pentyl] 2-methylpropanoate |
| 520958 | 1-(2,4-dimethylphenyl)-2,4-dimethylbenzene |
| 6041429 | E)-3-(2,4-dimethoxyphenyl)-1-(2-hydroxyphenyl)prop-2-en-1-one |
| 91742720 | bis[[(3-ethylphenoxy)-dimethylsilyl]oxy]-dimethylsilane |
| 5370325 | bis(2-ethylhexyl) (E)-but-2-enedioate |
| 574439 | 2-(hydroxymethyl)-2-methylpyrrolidine-1-carbaldehyde |
| 567717 | 2-benzyl-1-morpholin-4-yl-3-phenylpropan-2-ol |
| 5370158 | (E)-3-(2,2,6-trimethyl-7-oxabicyclo[4.1.0]heptan-1-yl)prop-2-enal |
| 21630 | 2-ethylhexyl 3-(4-methoxyphenyl)prop-2-enoate |
| 137353 | 3-ethyl-3-[4-(hydroxyamino)phenyl]piperidine-2,6-dione |
| 558880 | (5-oxooxolan-2-yl)methyl 3,5-dinitrobenzoate |
| 73219 | 3,5-ditert-butyl-4-hydroxybenzaldehyde |
| 141033 | 3,6,6-trimethylcyclohex-2-en-1-one |
| 91731726 | (4-trimethylsilyloxybenzoyl) 4-trimethylsilyloxybenzoate |
| 575987 | 5H-cyclopenta[b]pyridine |
| 7410 | 1-phenylethanone |
| 91713521 | 1-O-butyl 6-O-propan-2-yl hexanedioate |
| 1303 | 2-hydroxy-2-phenylpropanoic acid |
| 9231 | azulene |
| 240 | benzaldehyde |
| 7602 | 1,3,5-triethylbenzene |
| 12160 | 1-ethyl-4-methylbenzene |
| 998 | 2-phenylacetaldehyde |
| 12053 | 2-phenylpropan-2-ol |
| 7147 | 1-phenylpropan-1-ol |
| 637516 | (1R)-1-phenylethanol |
| 103653 | 2-methylbutyl benzoate |
| 90232 | ethyl 4-ethoxybenzoate |
| 3102 | diphenylmethanone |

Table S2. The common PubChem CID and IUPAC Name lists for each disease used in this study are provided below. (continued)

| PubChem CID | IUPAC Name |
| --- | --- |
| 7222 | 1,3-benzothiazole |
| 244 | phenylmethanol |
| 8343 | bis(2-ethylhexyl) benzene-1,2-dicarboxylate |
| 85781 | bis(2-ethylhexyl) carbonate |
| 9268 | cyclododecane |
| 7874 | 2,2,4,4,6,6,8,8,10,10,12,12,14,14-tetradecamethyl-1,3,5,7,9,11,13-heptaoxa-2,4,6,8,10,12,14-heptasilacyclotetradecane |
| 11170 | 2,2,4,4,6,6,8,8,10,10,12,12,14,14,16,16-hexadecamethyl-1,3,5,7,9,11,13,15-octaoxa-2,4,6,8,10,12,14,16-octasilacyclohexadecane |
| 11169 | 2,2,4,4,6,6,8,8-octamethyl-1,3,5,7,2,4,6,8-tetraoxatetrasilocane |
| 10914 | 2,2,4,4,6,6-hexamethyl-1,3,5,2,4,6-trioxatrisilinane |
| 15600 | decane |
| 8194 | dodecanal |
| 75084 | dodecyl prop-2-enoate |
| 8133 | 2-butoxyethanol |
| 31236 | 2-phenoxyethanol |
| 22568 | 2-phenoxyethyl acetate |
| 23229 | 1-(3-acetylphenyl)ethanone |
| 7500 | ethylbenzene |
| 2758 | 1,3,3-trimethyl-2-oxabicyclo[2.2.2]octane |
| 19602 | 2-pentylfuran |
| 160669 | (2S)-5-amino-2-(methylamino)-5-oxopentanoic acid |
| 41209 | 2,6,10,15-tetramethylheptadecane |
| 16663 | 4-ethylheptane |
| 11006 | hexadecane |
| 557859 | 3,7-dimethyloct-7-en-3-ol |
| 4133 | methyl 2-hydroxybenzoate |
| 13544681 | N-methyl-N-[2-(methylamino)ethyl]benzamide |
| 537332 | 5-ethyl-2-methyloctane |
| 9602988 | methyl (Z)-N-hydroxybenzenecarboximidate |
| 2879 | 4-methylphenol |
| 21205 | methyl 14-methylpentadecanoate |
| 460 | 2-methoxyphenol |
| 8814 | 4-(2,4,4-trimethylpentan-2-yl)phenol |
| 91726212 | 4-O-[2-(2-chlorophenoxy)ethyl] 1-O-ethyl butanedioate |
| 11230 | 4-methyl-1-propan-2-ylcyclohex-3-en-1-ol |
| 12388 | tridecane |
| 31355 | tris(2-methylpropyl) phosphate |
| 14257 | undecane |
